# Non-pyroptotic caspase-11 activity regulates osteoclastogenesis and pathological bone loss

**DOI:** 10.1038/s41418-025-01596-3

**Published:** 2025-10-22

**Authors:** Xianyu Piao, Ju Han Song, Jung-Woo Kim, Seung-Hee Kwon, Sin-Hye Oh, Sangita Sharma, Suk-Gyun Park, Zhao Wang, Zhiyu Fang, Je-Hwang Ryu, Nacksung Kim, Jeong-Tae Koh

**Affiliations:** 1 Department of Pharmacology and Dental Therapeutics, School of Dentistry, Chonnam National University, Gwangju, Republic of Korea; 2 Hard-tissue Biointerface Research Center, School of Dentistry, Chonnam National University, Gwangju, Republic of Korea; 3 Department of Pharmacology, Chonnam National University Medical School, Gwangju, Republic of Korea

**Keywords:** Cell biology, Diseases, Inflammation

## Abstract

Osteoclasts are essential for bone remodeling; however, their hyperactivity leads to pathological bone loss. While inflammasome-activated caspases are known to influence osteoclastogenesis, the role of caspase-11, beyond its conventional function in pyroptosis, remains unclear. Here, we identified caspase-11 as a pivotal regulator of RANKL-induced osteoclast differentiation. Caspase-11 expression and activity were elevated in bone tissues exhibiting excessive resorption and in RANKL-stimulated bone marrow-derived macrophages. Unlike inflammasome activation, RANKL-induced caspase-11 did not trigger typical inflammasome-associated inflammatory responses. Caspase-11 knockout mice displayed increased bone mass and resistance to RANKL-induced bone resorption; in parallel, genetic or pharmacological inhibition of caspase-11 impaired osteoclast differentiation in vitro. Notably, mechanistic studies revealed that RANKL-activated caspase-11 translocates to the nucleus, where it cleaves and inactivates poly(ADP-ribose) polymerase 1 (PARP1), a transcriptional repressor of osteoclastogenesis. In addition, using the caspase-11 inhibitor, VX-765, substantially reduced ovariectomy-induced bone loss. These findings collectively reveal a novel, non-inflammatory function of caspase-11 in osteoclastogenesis, positioning it as a promising therapeutic target for osteolytic diseases.

## Introduction

Bone remodeling is a complex, continuous process involving the coordinated actions of osteoclasts and osteoblasts to maintain skeletal integrity throughout life [1]. Dysregulation of this process leads to osteoporosis, a debilitating condition characterized by increased bone resorption, decreased bone mass, and a significantly increased fracture risk. Osteoporosis is a global health problem affecting millions of individuals and is projected to increase with aging populations and changing lifestyles [2]. Given the significant burden of osteoporosis, elucidating the underlying mechanisms is critical for developing novel therapeutic strategies for treating and preventing this disease.

Osteoclasts, the cells responsible for bone resorption, arise from the differentiation of mononuclear pre-osteoclasts. This process is critically regulated by receptor activator of nuclear factor κB ligand (RANKL) and macrophage colony-stimulating factor (M-CSF) signaling [3]. The binding of RANKL to its receptor, RANK, on pre-osteoclasts initiates signaling pathways involving nuclear factor-κB (NF-κB) and mitogen-activated protein kinases (MAPKs), culminating in the activation of nuclear factor of activated T-cells, cytoplasmic 1 (NFATc1), a master regulator of osteoclast differentiation. NFATc1 activation promotes the expression of key osteoclast-related genes such as *tartrate-resistant acid phosphatase* (*Trap*) and *cathepsin K* (*Ctsk*) [4]. This process is intricately modulated by chromatin remodeling mechanisms, including methylation, acetylation, and poly(ADP)-ribosylation (PARylation) [5–7].

Inflammatory caspases (caspase-1, -4, -5, and -11), a subset of cysteine-dependent aspartate-specific proteases, are primarily responsible for inflammatory responses. These enzymes promote the maturation of proinflammatory cytokines, such as interleukin (IL)-1β and IL-18, and cleave gasdermin D (GSDMD), triggering pyroptotic cell death [8–10]. Caspase-1 is predominantly activated by canonical inflammasomes, such as the NLRP3 inflammasome, in response to pathogen-associated molecular patterns (PAMPs) and damage-associated molecular patterns (DAMPs) [11]. Conversely, caspase-11 (or caspase-4/-5 in humans) orchestrates the non-canonical inflammasome pathway [12]. Activation of caspase-11 requires a priming phase, driven by factors such as bacterial lipopolysaccharide (LPS), interferons (IFNs), IL-1, and high mobility group box 1 [13, 14]. Unlike the canonical pathway, caspase-11 directly binds to cytosolic LPS, leading to oligomerization and autoproteolysis, thus bypassing traditional inflammasome components [12, 15, 16]. Excessive activation of caspase-11 has been strongly associated with immune-related diseases, particularly sepsis [12, 17].

While caspase-11 is primarily known for its role in pyroptosis, recent studies suggest its involvement in non-inflammatory cellular processes [14, 18]. Our previous study demonstrated that NLRP3 inflammasome-related caspase-1 contributes to age-related alveolar bone loss through inflammation-dependent and independent regulation of osteoclast differentiation [19]. Thus, this present study aimed to investigate the role of caspase-11 in osteoclastogenesis. Our results indicate that caspase-11 plays a unique role in the initiation of osteoclast differentiation in vitro, distinct from traditional inflammasome activation. Furthermore, we demonstrate that genetic ablation and pharmacological inhibition of caspase-11 preserve bone integrity in osteoporotic conditions. This research uncovers a novel non-pyroptotic function of caspase-11 in osteoclastogenesis and suggests new therapeutic avenues to mitigate osteoclast-associated bone loss.

## Results

### Caspase-11 is upregulated in experimental models of bone loss

**Fig. 1:**
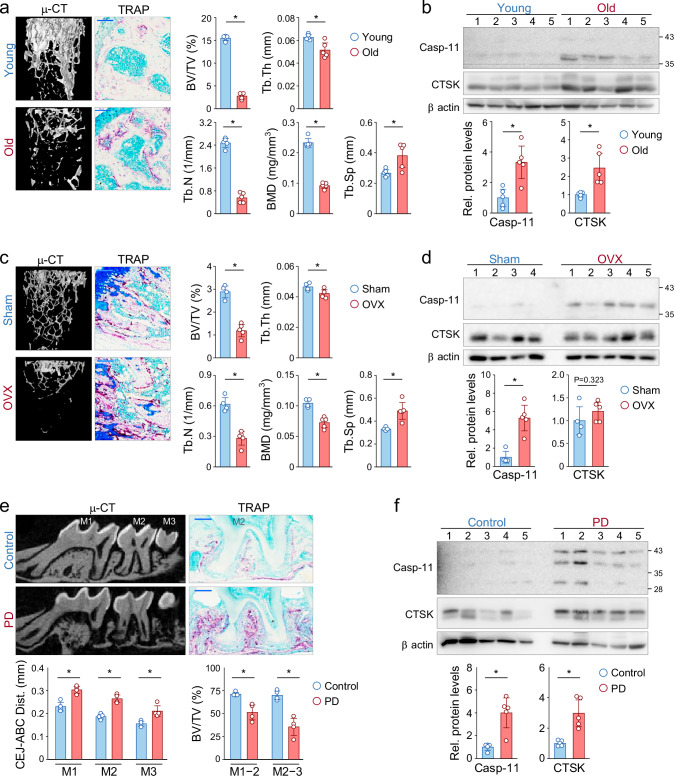
Caspase-11 is upregulated in bone tissues from experimental bone loss models. **a** Representative 3D μ-CT images and TRAP-stained sections of distal femoral trabecular bone from young (6 months; n = 5) and aged (29 months; n = 5) mice. Morphometric parameters, including BV/TV (bone volume/tissue volume), Tb.Th (trabecular thickness), Tb.N (trabecular number), BMD (bone mineral density), and Tb.Sp (trabecular separation), are presented as the mean ± SD. Scale bar, 0.1 mm for TRAP-stained sections. **b** Western blot analysis of caspase-11 and cathepsin K (CTSK) expression in tibiae of young and aged mice. **c** Representative 3D μ-CT images and TRAP-stained sections of distal femoral trabecular bone from sham-operated (n = 4) and ovariectomized (OVX; n = 5) mice. Scale bar, 0.1 mm for TRAP-stained sections. **d** Western blot analysis of caspase-11 and CTSK expression in femurs of sham-operated or OVX mice. **e** Representative 2D μ-CT images and TRAP-stained sections of maxillary alveolar bone from control mice (n = 4) and mice with silk ligature-induced periodontitis (PD; n = 4). The cementoenamel junction to alveolar bone crest (CEJ-ABC) distance was measured at three buccal sites: the distal root of the first molar (M1), both roots of the second molar (M2), and the third molar (M3). BV/TV measurements were performed on the alveolar bone between M1 and M2 (M1 − 2) and between M2 and M3 (M2 − 3). Scale bar, 0.2 mm for TRAP-stained sections. **f** Western blot analysis of caspase-11 and CTSK expression in alveolar bone from control and PD mice. Protein levels were quantified using ImageJ software and normalized to β-actin. Data are expressed as fold change relative to control and presented as mean ± SD.

To investigate the involvement of caspase-11 in bone loss, we examined its expression in three experimental models: aging, ovariectomy (OVX), and periodontitis. In the aging model, micro-computed tomography (μ-CT) analysis confirmed significant bone mass reduction, accompanied by an increased number of tartrate-resistant acid phosphatase (TRAP)-positive osteoclasts (Fig. 1a). Western blot analysis revealed a statistically significant increase in caspase-11 protein (p43/p38 forms) in femoral bone tissue, correlating with increased expression of the osteoclast marker CTSK (Fig. 1b). In the OVX model, μ-CT analysis showed substantial trabecular bone loss in OVX-treated mice, consistent with an increase in TRAP-positive osteoclasts compared to sham-operated controls (Fig. 1c). Western blot analysis confirmed a significant upregulation of caspase-11 (p38 form) in the femurs of OVX-treated mice, whereas its expression was barely detectable in sham-operated controls. While CTSK levels also increased modestly, caspase-11 upregulation exhibited a stronger correlation with osteoclastic bone resorption (Fig. 1d). In the periodontitis model, ligature-induced alveolar bone loss was observed via μ-CT analysis, along with increased TRAP staining of osteoclasts in affected regions (Fig. 1e). Consistently, Western blot analysis demonstrated elevated expression of caspase-11 and CTSK in alveolar bone samples (Fig. 1f). Collectively, these findings suggest that caspase-11 upregulation is strongly associated with osteoclast-mediated bone loss across multiple pathological conditions.

### Caspase-11 expression and activity increase during RANKL-induced osteoclast differentiation

**Fig. 2:**
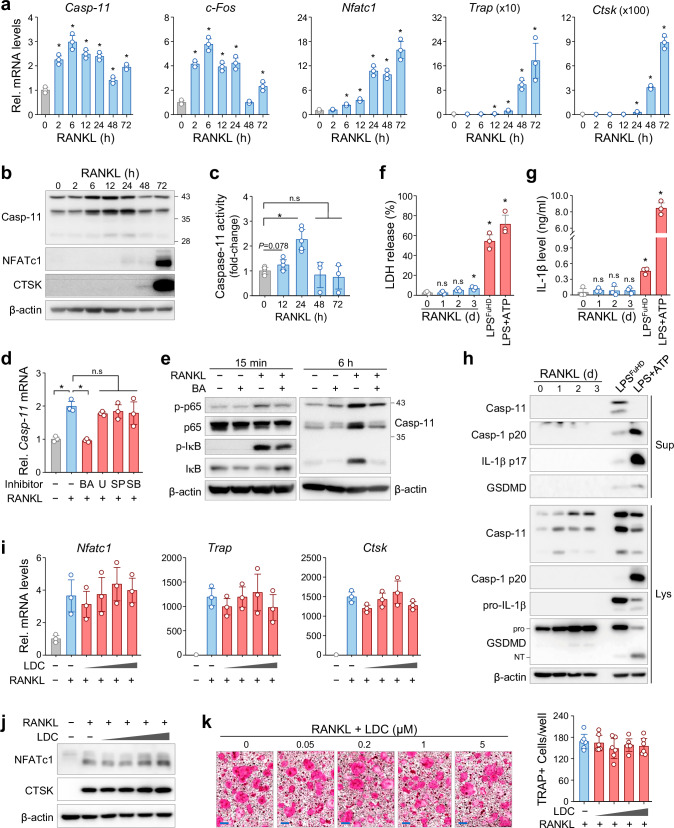
RANKL induces caspase-11 activation independent of pyroptosis. **a**–**c** Bone marrow-derived macrophages (BMMs) were treated with RANKL for the indicated time points. **a** qRT-PCR analysis of caspase-11 and osteoclast marker mRNA levels. Data are shown as fold change relative to untreated controls (mean ± SD, n = 3). **b** Western blot analysis of caspase-11 and osteoclast marker protein levels, with β-actin as an internal control. **c** Caspase-11 enzymatic activity was measured in whole-cell extracts. Data are expressed as fold changes relative to untreated samples and presented as the mean ± SD (n = 3). **d** BMMs were pretreated with specific inhibitors for 15 min, followed by RANKL stimulation for 6 h. *Caspase-11* mRNA levels were analyzed by qRT-PCR and normalized to untreated controls. Data are presented as the mean ± SD (n = 3). BA, Bay11-7082 (NF-κB); U, U0126 (ERK); SP, SP600125 (JNK); and SB, SB202190 (p38). **e** BMMs were pretreated with BA for 30 min, followed by stimulation with RANKL for the indicated periods. Western blot analysis assessed caspase-11 protein levels, with p-p65 and p-IκB levels used as positive controls. **f**–**h** BMMs were treated with RANKL for the indicated time points. For positive controls of non-canonical and canonical inflammasome activation, cells were transfected with LPS (LPS^FuHD^) or treated with LPS and ATP (LPS + ATP). **f** Cytotoxicity was measured by LDH release assay. **g** IL-1β levels in culture supernatants were measured by ELISA. *P < 0.05 *vs*. untreated control. n.s, not significant. **h** Levels of inflammasome-related proteins in conditioned supernatants (Sup) and whole cell lysates (Lys) were analyzed by Western blotting. **i**–**k** BMMs were treated with RANKL in the presence or absence of LDC7559 (LDC; 0.05, 0.2, 1, 5 μM) for 3 days. Osteoclast differentiation was determined by qRT-PCR (**i**), Western blotting (**j**), and TRAP staining (**k**).

Given the observed increase in caspase-11 levels in osteoporotic bone (Fig. 1), we next investigated its expression dynamics during RANKL-induced osteoclast differentiation. In bone marrow-derived macrophages (BMMs), RANKL stimulation significantly increased caspase-11 mRNA and protein expression, coinciding with upregulation of key osteoclastogenic markers, including c-Fos, Nfatc1, Trap, and Ctsk (Fig. 2a, b). Interestingly, caspase-11 expression peaked at early differentiation stages, preceding the maximal expression of most osteoclast markers. Moreover, its expression increased dose-dependently with RANKL stimulation, in parallel with *c-Fos* upregulation and NF-κB p65 phosphorylation (Supplementary Fig. 1). Enzymatic activity assays further confirmed that caspase-11 activity significantly increased during early osteoclastogenesis, consistent with its expression pattern (Fig. 2c).

To ascertain the upstream signaling pathway involved, we treated with BMMs with specific inhibitors targeting NF-κB (Bay11-7082), ERK (U0126), JNK (SP600125), and p38 (SB202190). Notably, only NF-κB inhibition effectively suppressed RANKL-induced caspase-11 expression (Fig. 2d). Western blot analysis confirmed that NF-κB blockage led to reduced caspase-11 protein levels, underscoring the critical role of the RANKL/RANK/NF-κB axis in caspase-11 induction (Fig. 2e).

### RANKL-induced caspase-11 upregulation is independent of inflammasome activation

To determine whether RANKL-induced caspase-11 upregulation involves inflammasome activation, we examined general pyroptosis markers, such as lactate dehydrogenase (LDH) release and IL-1β secretion. Unlike LPS transfection (non-canonical inflammasome activation) or LPS plus ATP treatment (canonical inflammasome activation), which resulted in significant LDH release, RANKL treatment exhibited minimal release by day 3 (Fig. 2f). Similarly, IL-1β secretion was negligible in RANKL-treated cells, unlike inflammasome-activating conditions (Fig. 2g). Western blot analysis of culture supernatants further confirmed the absence of inflammasome-associated markers (IL-1β, caspase-1 p20, and cleaved-GSDMD) despite caspase-11 upregulation (Fig. 2h). To further assess whether the inflammasome-mediated pyroptosis plays a functional role in osteoclast differentiation, we employed LDC7559, a selective inhibitor of GSDMD pore formation. Based on preliminary dose-finding experiments (Supplementary Fig. 2), we used a concentration that effectively suppressed GSDMD activation. Under these conditions, LDC7559 treatment had negligible effects on RANKL-induced osteoclastogenesis, as demonstrated by qRT-PCR and Western blot analyses of osteoclast differentiation markers and TRAP staining (Fig. 2i−k). Collectively, these results suggest that RANKL-induced caspase-11 expression occurs independently of inflammasome activation and pyroptosis, supporting the idea that caspase-11 plays a distinct, non-inflammatory role in osteoclast differentiation.

### Caspase-11 positively regulates osteoclast differentiation

**Fig. 3:**
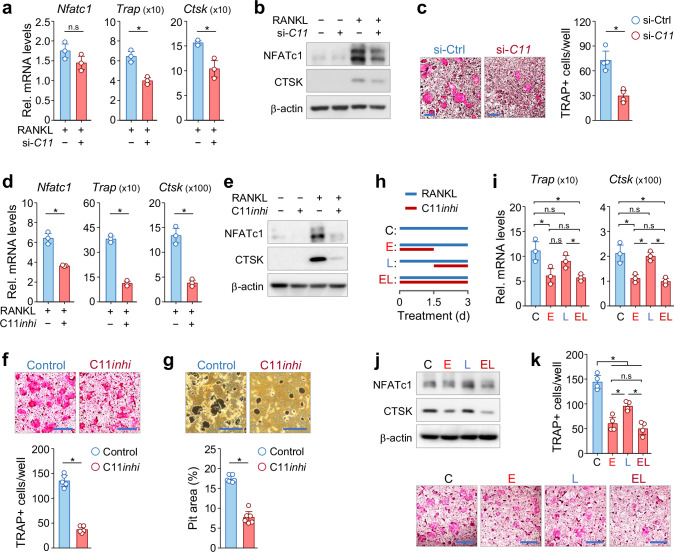
Caspase-11 positively regulates osteoclast differentiation. **a**–**c** Bone marrow-derived macrophages (BMMs) were transfected with control siRNA (si-Ctrl) or caspase-11 siRNA (si-*C11*) and stimulated with RANKL for 3 days. **a** qRT-PCR analysis of osteoclast marker mRNA levels. Data are expressed as the fold change relative to si-Ctrl and shown as the mean ± SD (n = 3). **b** Western blot analysis of osteoclast marker protein levels, with β-actin as an internal control. **c** TRAP staining was performed to assess osteoclast formation and number. Scale bar, 500 μm. **d**–**g** BMMs were cultured with RANKL in the presence or absence of the caspase-11 inhibitor Ac-LEVD-CHO (C11*inhi*, 50 μM) for 3 days. **d** qRT-PCR analysis of osteoclast marker mRNA levels. Results are expressed as the fold change relative to untreated control and presented as the mean ± SD (n = 3). **e** Western blot analysis of osteoclast marker protein levels, with β-actin as an internal control. **f** Osteoclast formation was assessed by TRAP staining. Scale bar, 500 μm. **g** Bone resorption activity was measured using a pit formation assay. **h**–**k** BMMs were treated with Ac-LEVD-CHO (C11*inhi*) in the presence of RANKL, as indicated. **h** Schematic diagram of the experimental design. **i** qRT-PCR analysis of osteoclast marker mRNA levels. Results are expressed as the fold change relative to untreated controls and presented as the mean ± SD (n = 3). **j** Western blot analysis of osteoclast marker proteins, with β-actin as a loading control. **k** Osteoclast formation and number were evaluated by TRAP staining. Scale bar, 500 μm.

To assess the functional role of caspase-11 in osteoclastogenesis, we performed siRNA-mediated knockdown in BMMs. The knockdown efficiency was confirmed by quantitative real-time reverse transcription-polymerase chain reaction (qRT-PCR) and Western blot analyses (Supplementary Fig. 3a, b). Caspase-11 knockdown led to a marked reduction in the expression of key osteoclast markers, including Trap and Ctsk, and the master regulator NFATc1 (Fig. 3a, b). TRAP staining further confirmed a significant decrease in osteoclast formation (Fig. 3c). Similarly, pharmacological inhibition of caspase-11 using Ac-LEVD-CHO suppressed RANKL-induced osteoclastogenesis. Inhibitor treatment significantly reduced caspase-11 activity and osteoclast marker expression (Supplementary Fig. 3c, d and Fig. 3d, e), as well as osteoclast formation and hydroxyapatite resorption (Fig. 3f, g).

Given the dynamic expression of caspase-11 during osteoclast differentiation (Fig. 2), we next investigated the critical time points for its function. Time-course experiments revealed that caspase-11 inhibition during the early differentiation phase (E) significantly impaired osteoclastogenesis, with effects comparable to continuous inhibition (EL). In contrast, inhibition during the later phase (L) had minimal impact (Fig. 3h−k). These results indicate that caspase-11 is crucial for the initiation of RANKL-induced osteoclastogenesis, expanding its functional repertoire beyond non-canonical inflammasome activation.

### Genetic ablation of caspase-11 attenuates RANKL-induced bone loss in vivo

**Fig. 4:**
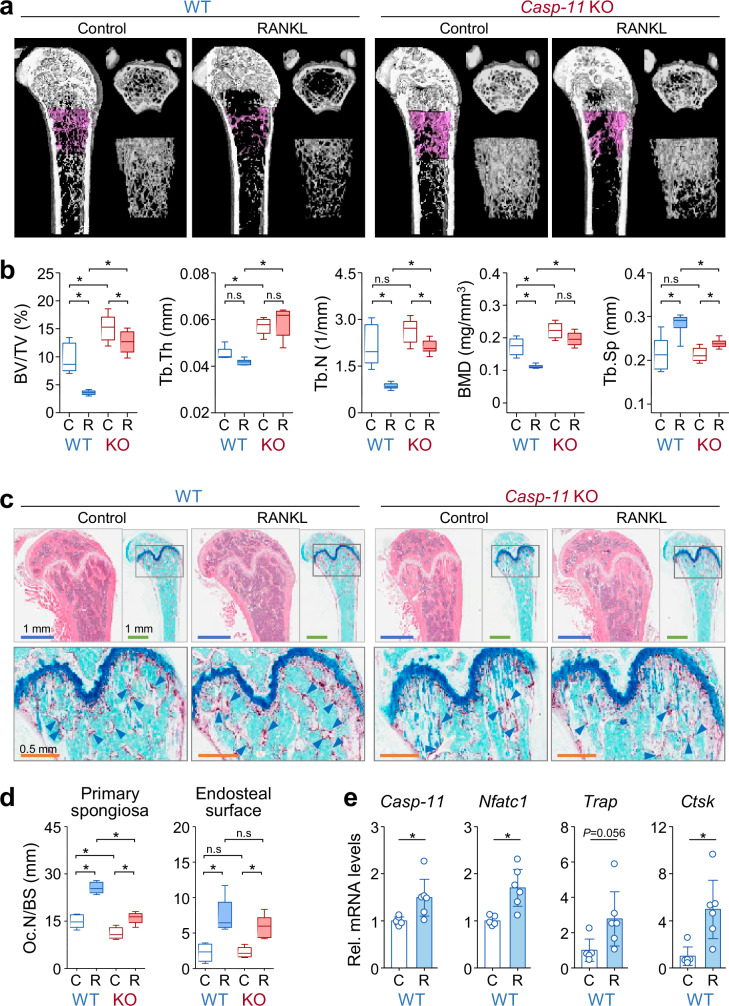
Caspase-11 deficiency attenuates RANKL-induced bone loss in vivo. **a** Representative 3D μ-CT images of the distal femur of wild-type (WT) and *caspase-11* knockout (*Casp-11* KO) mice after vehicle or RANKL injection. Longitudinal (left), transverse (upper right), and coronal (lower right) views of trabecular bone at the distal metaphysis are shown. **b** Quantification of trabecular bone morphometric parameters, including bone volume/tissue volume (BV/TV), trabecular thickness (Tb.Th), trabecular number (Tb.N), bone mineral density (BMD), and trabecular separation (Tb.Sp). Data are presented as box-plots with median (horizontal line) and minimum and maximum values (whiskers). Vehicle-treated groups: n = 5; RANKL-treated groups: n = 6. n.s, not significant. **c** Representative images of hematoxylin and eosin (H&E, upper left) and TRAP staining of the distal femur. Magnified TRAP-stained images (lower) show osteoclasts (arrowheads) on the primary spongiosa. **d** Quantification of TRAP-positive osteoclasts is expressed as the number of osteoclasts per bone surface (Oc.N/BS). Box-plots as in (**b**). **e** qRT-PCR analysis of caspase-11 and osteoclast-related gene expression in whole bone tissues from WT mice. Data are expressed as the mean ± SD (n = 3).

To further evaluate the in vivo role of caspase-11 in osteoclastogenesis, we injected RANKL into caspase-11 wild-type and knockout mice and assessed bone mass alterations. μ-CT analysis revealed that caspase-11 knockout mice exhibited significantly higher baseline trabecular and cortical bone mass compared to wild-type controls (Fig. 4a, b and Supplementary Fig. 4). Following RANKL administration, both groups experienced bone loss; however, caspase-11 knockout mice showed significantly attenuated reductions. In the femur, trabecular bone volume fraction (BV/TV) decreased by 63% in wild-type mice but only by 16% in knockout mice. Trabecular thickness (Tb.Th) exhibited an 8% decrease in wild-type mice and a 3% increase in knockout mice. Trabecular number (Tb.N) decreased by 60% in wild-type mice but only by 20% in knockout mice. Similarly, bone mineral density (BMD) decreased by 35% in wild-type mice and 12% in knockout mice, whereas trabecular separation (Tb.Sp) increased by 33% in wild-type mice compared to 12% in knockout mice (Fig. 4a, b). Comparable protective effects were observed in vertebral trabecular bone (Supplementary Fig. 4a, b). Meanwhile, femoral cortical bone remained largely unaffected by RANKL administration (Supplementary Fig. 4c, d).

Histological analysis corroborated these findings. Hematoxylin and eosin (H&E) staining confirmed increased trabecular bone mass in caspase-11 knockout mice, while TRAP staining revealed significantly fewer TRAP-positive osteoclasts in the primary spongiosa compared to wild-type controls (Fig. 4c, d). Although osteoclast numbers increased following RANKL injection, the magnitude of the increase was significantly lower in knockout mice (Fig. 4c, d). qRT-PCR analysis of femoral bones from wild-type mice further confirmed RANKL-induced upregulation of *caspase-11*, accompanied by elevated expression of *Nfatc1*, *Trap*, and *Ctsk* (Fig. 4e).

To determine whether increased bone mass in caspase-11 knockout mice resulted from cumulative effects during growth, we examined femoral bones from 4-week-old mice. Despite no difference in body weight or growth plate thickness (Supplementary Fig. 5a, b), H&E staining and μ-CT analysis revealed significantly higher trabecular bone mass in the knockout mice at this early age (Supplementary Fig. 5c−e). These findings suggest that caspase-11 plays a crucial role in RANKL-induced osteoclastogenesis and continuous bone remodeling throughout life.

### Caspase-11 deficiency impairs RANKL-induced osteoclastogenesis in vitro

**Fig. 5:**
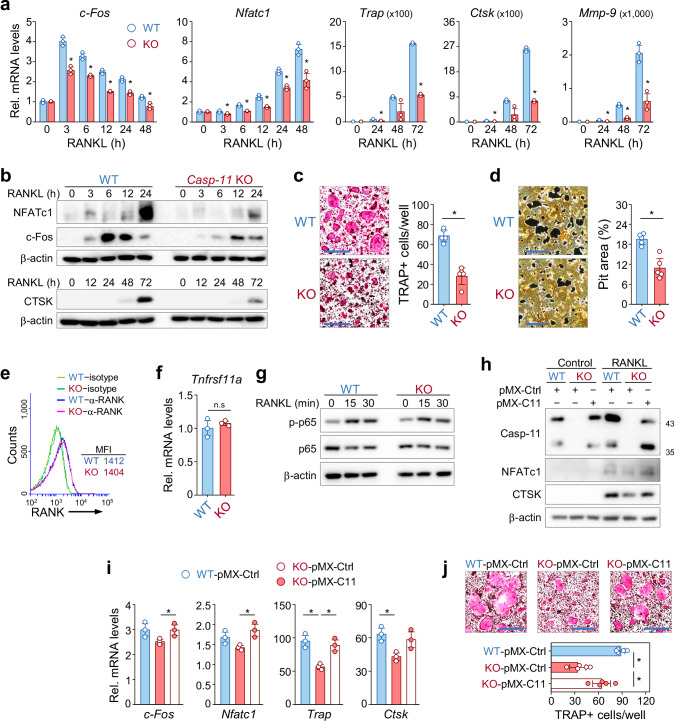
Genetic ablation of caspase-11 inhibits osteoclastogenesis. **a**, **b** Bone marrow-derived macrophages (BMMs) from wild-type (WT) and caspase-11 knockout (KO) mice were stimulated using RANKL for the indicated times. **a** qRT-PCR analysis of osteoclast-related gene expression. Data are expressed as the mean ± SD (n = 3). **b** Western blot analysis of osteoclast marker proteins, with β-actin as an internal control. Representative images of TRAP staining (**c**) and pit formation assay (**d**) in RANKL-treated BMMs from WT and KO mice. Quantification of TRAP-positive cells and pit area is presented as the mean ± SD. Scale bar, 500 μm. **e** Flow cytometric analysis of RANK expression on cell surface of WT and caspase-11 KO BMMs. Representative histograms are shown, with mean fluorescence intensity (MFI) obtained from three independent experiments. **f** qRT-PCR analysis of *Tnfrsf11a* (RANK) mRNA levels in WT and KO BMMs. Data are expressed as the mean ± SD (n = 3). n.s, not significant. **g** Western blot analysis of NF-κB activation in WT and KO BMMs, with β-actin as internal control. **h**–**j** BMMs from WT and *caspase-11* KO mice were infected with retrovirus expressing either a control vector (pMX–Ctrl) or caspase-11 (pMX−C11) and stimulated with or without RANKL for 72 h. **h** Western blot analysis of caspase-11 and osteoclast markers, with β-actin as an internal control. **i** qRT-PCR analysis of osteoclast-related gene expression. **j** Representative TRAP-stained images and quantification of TRAP-positive cells. Data are expressed as the mean ± SD. Scale bar, 500 μm.

To further explore the mechanism underlying the reduced bone loss in caspase-11 knockout mice, we examined RANKL-induced osteoclastogenesis in vitro using BMMs from wild-type and caspase-11 knockout mice. RANKL stimulation significantly upregulated osteoclast markers (c-Fos, Nfatc1, Trap, Ctsk, and Mmp-9) in wild-type cells; however, this response was markedly blunted in caspase-11-deficient BMMs (Fig. 5a, b). Consistently, TRAP staining and pit formation assays confirmed significant defects in osteoclast formation and bone resorptive activity in caspase-11-deficient cells (Fig. 5c, d).

To determine whether impaired osteoclastogenesis resulted from defects in RANKL/RANK signaling, we examined RANK expression and downstream signaling in wild-type and knockout BMMs. Flow cytometry and qRT-PCR analyses revealed no significant differences in RANK surface expression or mRNA levels between the two groups (Fig. 5e, f). Furthermore, RANKL-induced phosphorylation of NF-κB p65, a key downstream effector, was comparable in both wild-type and knockout cells (Fig. 5g), indicating that the RANKL/RANK pathway remains intact.

To confirm that the observed defects were directly attributable to caspase-11 loss, we reintroduced caspase-11 into knockout BMMs via retroviral transduction. Western blot analysis confirmed successful restoration of caspase-11 expression (Fig. 5h). Notably, caspase-11-reconstituted cells exhibited a significant recovery in osteoclast differentiation upon RANKL treatment (Fig. 5h−j). These findings establish caspase-11 as a critical regulator of RANKL-induced osteoclastogenesis, reinforcing its essential role in bone resorption.

### Caspase-11 mediates PARP1 cleavage to promote osteoclast differentiation

Previous studies have highlighted the pivotal role of poly(ADP-ribose) polymerase-1 (PARP1) in osteoclast differentiation, acting through transcriptional regulation via PARylation and its proteolytic degradation by inflammatory caspases [6, 20]. To examine the involvement of caspase-11 in PARP1 regulation during osteoclast differentiation, we first confirmed the inhibitory role of PARP1. Knockdown of PARP1 via siRNA significantly increased the formation of TRAP-positive cells and upregulated the expression of osteoclast markers (TRAP, CTSK, and NFATc1) following RANKL treatment (Supplementary Fig. 6a−c). Similarly, pharmacological inhibition of PARP1 with rucaparib, a specific PARP1 inhibitor, led to increased osteoclast marker expression and osteoclast formation, along with reduced PAR levels (Supplementary Fig. 6d−f). These results confirm that PARP1 functions as a negative regulator of osteoclast differentiation, consistent with previous findings.

**Fig. 6:**
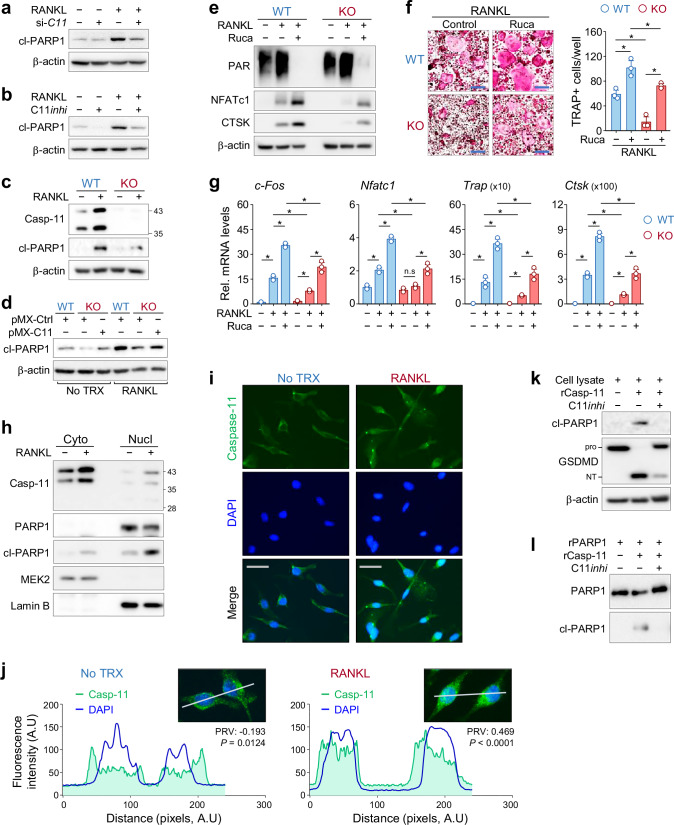
Caspase-11 regulates PARP1 proteolytic processing, a suppressor of osteoclast differentiation. **a** Western blot analysis of cleaved PARP1 (cl-PARP1) in bone marrow-derived macrophages (BMMs) transfected with either a control siRNA or a *caspase-11* siRNA (si-*C11*) and then treated with RANKL for 24 h. β-actin was used as a loading control. **b** Western blot analysis of cl-PARP1 in BMMs treated with RANKL in the presence or absence of the caspase-11 inhibitor Ac-LEVD-CHO (C11*inhi*) for 24 h. **c** Western blot analysis of caspase-11 and cl-PARP1 in BMMs isolated from wild-type (WT) and *caspase-11* knockout (KO) mice following RANKL treatment for 24 h. **d** WT and KO BMMs were infected with a retrovirus containing either a control vector (pMX–Ctrl) or caspase-11 (pMX−C11). Then, the cells were treated with RANKL for 24 h, and cl-PARP1 levels were analyzed by Western blotting. **e**–**g** WT and KO BMMs were treated with RANKL for 3 days in the presence or absence of rucaparib (Ruca). **e** Western blot analysis of PAR, NFATc1, and CTSK. **f** Representative TRAP-stained images of osteoclasts with quantification of TRAP-positive cells (mean ± SD). Scale bar, 200 μm. **g** qRT-PCR analysis of osteoclast marker expression, normalized to the mean of the untreated WT control (mean ± SD, n = 3). **h** Western blot analysis of the cytosolic and nuclear fractions of BMMs treated with RANKL for 24 h. MEK2 and lamin B were used as loading controls for the cytosolic and nuclear fractions, respectively. **i** Immunofluorescence staining of BMMs treated with RANKL for 24 h. Caspase-11 was detected using a specific antibody and an Alexa 488-conjugated secondary antibody (green), nuclei were counterstained with DAPI (blue). Scale bars, 25 μm. **j** Quantitative analysis of caspase-11 and DAPI colocalization along the white line using ImageJ. Fluorescence intensity profiles are shown, and colocalization was evaluated by Pearson’s R value (PRV). **k** In vitro cleavage assay of PARP1 by recombinant caspase-11 in whole-cell lysates from BMMs with or without the caspase-11 inhibitor, Ac-LEVD-CHO (C11*inhi*). cl-PARP1 and GSDMD (positive control) were detected by Western blotting. NT, N-terminal fragment. **l** Recombinant full-length PARP1 was incubated with recombinant caspase-11, with or without Ac-LEVD-CHO. cl-PARP1 was assessed by Western blotting.

We next investigated whether caspase-11 mediates PARP1 cleavage during osteoclast differentiation. Western blot analysis showed that RANKL stimulation induced PARP1 cleavage, as evidenced by the appearance of the p89 PARP1 fragment. However, both caspase-11 knockdown and pharmacological inhibition markedly reduced RANKL-induced PARP1 cleavage (Fig. 6a, b). Similarly, BMMs derived from caspase-11 knockout mice exhibited significantly diminished PARP1 cleavage upon RANKL stimulation (Fig. 6c). Restoration of caspase-11 expression in knockout BMMs via viral transduction successfully rescued PARP1 cleavage, confirming the essential role of caspase-11 in this process (Fig. 6d).

To determine whether inhibition of PARP1 could bypass the requirement for caspase-11 in osteoclast differentiation, we treated BMMs from wild-type and caspase-11 knockout mice with rucaparib in combination with RANKL. As expected, rucaparib treatment significantly enhanced RANKL-induced osteoclast differentiation in wild-type cells (Fig. 6e−g). Notably, blocking PARP1 activity in caspase-11-deficient BMMs partially rescued osteoclast differentiation (Fig. 6e−g). These findings suggest that caspase-11 functions as an upstream regulator of PARP1 cleavage, promoting osteoclast differentiation.

### Caspase-11 translocates to the nucleus and directly cleaves PARP1 upon RANKL stimulation

To elucidate the mechanism by which caspase-11 regulates the nuclear protein PARP1 during osteoclast differentiation, we examined its subcellular localization in cytoplasmic and nuclear fractions. Western blot analysis revealed that, under basal conditions, caspase-11 predominantly resides in the cytoplasm. Upon RANKL stimulation, however, caspase-11 accumulated in the nucleus, coinciding with increased PARP1 cleavage (Fig. 6h). This suggests that caspase-11 contributes to nuclear PARP1 processing during osteoclastogenesis. To assess the functional importance of nuclear caspase-11 directly, we introduced a nuclear localization signal (NLS)-tagged active caspase-11 into caspase-11 KO BMMs using a retroviral system (Supplementary Fig. 7a). Compared to full-length caspase-11, NLS-tagged caspase-11 exhibited enhanced nuclear localization and significantly increased PARP1 cleavage, as confirmed by Western blot analysis of whole lysates and subcellular fractions (Supplementary Fig. 7b, c). These results provide evidence that the nuclear localization of caspase-11 facilitates its proteolytic activity toward PARP1, supporting its role in RANKL-induced osteoclast differentiation. To corroborate these findings, we performed immunofluorescence staining in BMMs. Under basal conditions, caspase-11 was primarily localized in the cytoplasm. Following RANKL stimulation, however, caspase-11 was clearly detected in the nucleus (Fig. 6i, j), which further supports its nuclear involvement in PARP1 regulation.

Similar results were obtained using RAW 264.7 cells, which differentiate into osteoclasts upon RANKL treatment. In these cells, caspase-11 was upregulated and PARP1 cleavage occurred (Supplementary Fig. 8a). Subcellular fractionation confirmed the nuclear localization of caspase-11, accompanied by PARP1 cleavage (Supplementary Fig. 8b). Immunofluorescence analysis also revealed increased nuclear translocation of caspase-11 upon RANKL treatment (Supplementary Fig. 8c, d), which further validates our observations in BMMs.

To determine whether caspase-11 directly cleaves PARP1, we conducted in vitro enzyme assays. Whole-cell lysates from naive BMMs were incubated with recombinant caspase-11. Western blot analysis using an antibody specific to the PARP1 cleavage site (Asp214) confirmed the production of the p89 PARP1 fragment. This cleavage was effectively blocked by the caspase-11 inhibitor Ac-LEVD-CHO (Fig. 6k). To exclude the possibility of indirect cleavage by other proteases, we next incubated recombinant full-length PARP1 with recombinant caspase-11. Direct cleavage at Asp214 was confirmed and was again fully inhibited by Ac-LEVD-CHO (Fig. 6l). Together, these results show that RANKL stimulation induces caspase-11 nuclear translocation, which directly cleaves PARP1 and regulates osteoclast differentiation.

### Targeting caspase-11 attenuates ovariectomy-induced bone loss

**Fig. 7:**
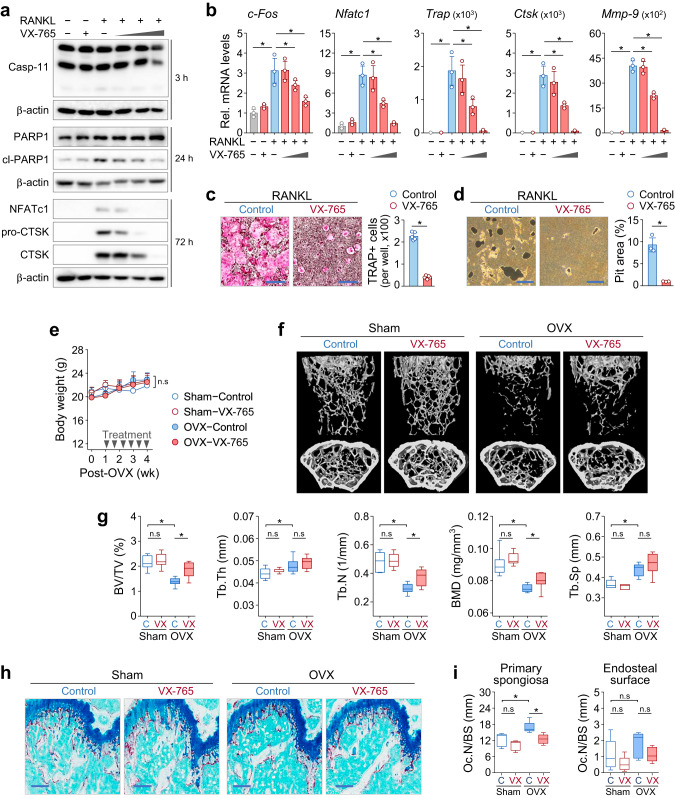
The caspase-11 inhibitor VX-765 attenuates osteoclastogenesis and prevents OVX-induced bone loss. **a**–**d** Bone marrow-derived macrophages (BMMs) were treated with RANKL in increasing concentrations of VX-765 (50, 100, and 200 μM; + indicates 200 μM). **a** Western blot analysis of the specified proteins at the indicated time points. **b** qRT-PCR analysis of osteoclast-related gene expression after 3 days of treatment. Data are expressed as mean ± SD (n = 3). Representative images of TRAP staining (**c**) and pit formation assay (**d**) with quantification of TRAP-positive osteoclasts and resorbed pit area, expressed as the mean ± SD. Scale bar, 500 μm. **e**–**i** Ovariectomized (OVX) or sham-operated mice were treated with VX-765 or vehicle (n = 8 per group). **e** Changes in body weight during the treatment period. **f** Representative 3D μ-CT images of the distal femur, showing coronal (top) and transverse (bottom) views of trabecular bone at the distal metaphysis. **g** μ-CT-based quantification of trabecular bone morphometric parameters, including bone volume/tissue volume (BV/TV), trabecular thickness (Tb.Th), trabecular number (Tb.N), bone mineral density (BMD), and trabecular separation (Tb.Sp). Box−plots represent the median (horizontal line within each box) and minimum/maximum values (whiskers). n.s, not significant. **h** Representative TRAP-stained images of distal femur sections. Scale bar, 0.2 mm. **i** Quantification of osteoclast numbers at the primary spongiosa and endosteal surfaces of the femurs, expressed as number of osteoclasts per bone surface (Oc.N/BS). n.s not significant.

To assess the therapeutic potential of caspase-11 inhibition, we selected VX-765 (Belnacasan), a selective inhibitor of interleukin-converting enzymes (ICEs), as a candidate caspase-11 inhibitor [21]. In vitro experiments were conducted to evaluate its effects on caspase-11 status and osteoclastogenesis. Western blot analysis confirmed that VX-765 effectively inhibited RANKL-induced caspase-11 activation and PARP1 cleavage (Fig. 7a). Additionally, VX-765 suppressed the expression of osteoclast differentiation markers in a dose-dependent manner (Fig. 7a, b), accompanied by a reduction in osteoclast formation and activity (Fig. 7c, d). These findings indicate that VX-765 impairs RANKL-induced osteoclastogenesis by inhibiting caspase-11 activity.

Next, we evaluated the in vivo efficacy of VX-765 using an OVX-induced osteoporosis model. Mice received intraperitoneal injection of VX-765 starting one week after OVX surgery and continuing for four weeks. No significant changes in body weight were observed, suggesting minimal drug toxicity and a favorable safety profile (Fig. 7e). μ-CT analysis of femurs revealed that OVX significantly reduced bone mass parameters, including BV/TV, Tb.N and BMD, in vehicle-treated mice compared to sham-operated controls. However, VX-765 treatment significantly attenuated these reductions (Fig. 7f, g), although no significant differences were observed in Tb.Th and Tb.Sp between the vehicle and VX-765 groups. TRAP staining of femur sections further demonstrated the efficacy of VX-765 in reducing osteoclast activity. OVX surgery markedly increased the number of osteoclasts per bone surface in the primary spongiosa but not in the endosteal surface. VX-765 treatment significantly reduced osteoclast numbers, confirming its role in suppressing osteoclast-mediated bone resorption (Fig. 7h, i). Furthermore, qRT-PCR analysis of bone tissue showed that VX-765 partially suppressed OVX-induced upregulation of osteoclast differentiation markers (Supplementary Fig. 9a). Western blot analysis of bone samples detected a modest increase in the caspase-11 p30 fragment following OVX surgery. However, contrary to our initial hypothesis, this increase persisted despite VX-765 administration (Supplementary Fig. 9b). Overall, these findings demonstrate that VX-765 attenuates OVX-induced bone loss by suppressing osteoclast differentiation and activity, supporting its potential as a therapeutic strategy for osteoporosis and other osteoclast-mediated bone diseases.

## Discussion

**Fig. 8:**
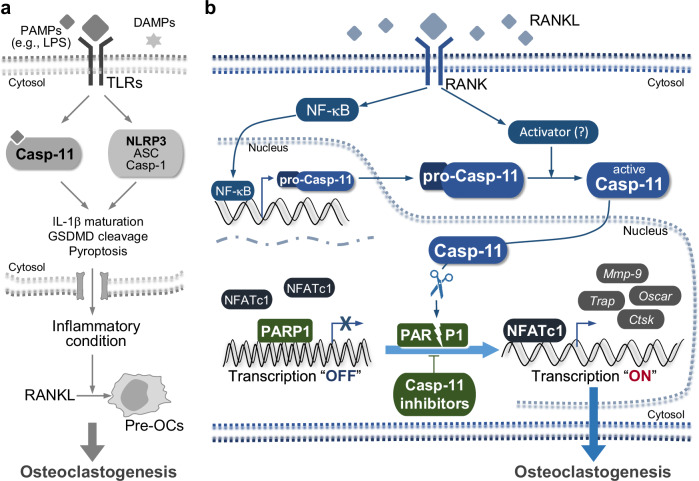
Proposed models for caspase-11-mediated regulation of osteoclastogenesis. **a** Indirect regulation via the non-canonical caspase-11 inflammasome. Caspase-11 indirectly promotes osteoclastogenesis by activating the non-canonical inflammasome. This activation facilitates pyroptosis and/or IL-1β secretion in cooperation with canonical inflammasome components, such as NLRP3. The resulting inflammatory milieu enhances osteoclast differentiation. **b** Direct regulation independent of inflammasome activation. In this pathway, RANKL stimulation upregulates the expression and activity of caspase-11 in pre-OCs, leading to its nuclear translocation. Once in the nucleus, caspase-11 cleaves PARP1, a suppressor of osteoclastogenesis, thereby directly promoting osteoclast differentiation. This pathway operates independently of the non-canonical inflammasome. Abbreviations: ASC apoptosis-associated speck-like protein containing a CARD, Ctsk cathepsin K, DAMPs damage-associated molecular patterns, GSDMD gasdermin D, Mmp-9 matrix metalloproteinase-9, NF-κB nuclear factor κB, NFATc1 nuclear factor of activated T-cells, cytoplasmic 1, NLRP3 NLR family pyrin domain containing 3, Oscar osteoclast-associated Ig-like receptor, PAMPs pathogen-associated molecular patterns, PARP1 poly (ADP-ribose) polymerase 1, Pre-OCs pre-osteoclasts, TLRs toll-like receptors, Trap tartrate-resistant acid phosphatase.

Caspase-11 was preferentially recognized for its role in non-canonical inflammasome activation through direct sensing of intracellular LPS. While its function in pyroptosis-mediated immune diseases is well established, its role in bone biology remains largely unexplored. Here, we identify caspase-11 as a novel regulator of osteoclastogenesis, independent of its inflammatory functions. Specifically, we demonstrate that caspase-11 directly cleaves PARP1, a suppressor of osteoclast differentiation, thereby promoting osteoclastogenesis (Fig. 8). These findings reveal an unrecognized function of caspase-11 in bone homeostasis and highlight its potential as a therapeutic target for osteoclast-mediated bone diseases such as osteoporosis.

We identified RANKL as an endogenous regulator of caspase-11 expression and activity. Caspase-11 levels were consistently elevated in bone tissues from animal models of osteoporosis associated with increased RANKL expression, including aging, menopause, and periodontitis [22–24]. Additionally, RANKL administration directly induced caspase-11 expression in bone tissue, resembling LPS-induced upregulation via NF-κB signaling in bacterial infection models [25]. However, unlike LPS, which triggers secondary amplification via type 1 IFNs, RANKL-induced caspase-11 expression occurs independently of IFN signaling (data not shown). This may explain the more moderate increase in caspase-11 expression compared to LPS stimulation.

In addition to upregulating caspase-11 expression, RANKL enhanced its enzymatic activity, as evidenced by the presence of its active forms (p38/p30) in the Western blot analysis and increased activity in the enzymatic assay. Notably, unlike classical non-canonical inflammasome activation, RANKL-induced caspase-11 activity did not lead to cytokine release or GSDMD-mediated pyroptosis. Instead, caspase-11 exhibited sublytic activity, promoting osteoclast differentiation without triggering cell death. This aligns with reports suggesting non-pyroptotic roles for caspase-11, such as facilitating cell migration [14, 18]. However, the precise mechanism of caspase-11 activation in osteoclastogenesis‒whether through homodimerization and autoproteolysis, or cleavage by an upstream protease‒remains unclear and warrants further investigation.

PARP1 is a nuclear enzyme involved in DNA repair, genome integrity, and transcriptional regulation via ADP-ribosylation [26]. Although PARP1 has been extensively studied as a therapeutic target in oncology [27], its role in osteoclastogenesis is also being recognized. PARP1 acts as a transcriptional repressor of genes related to osteoclasts, such as *Trap* and *brain-type cytoplasmic creatine kinase* [28, 29]. Upon RANKL stimulation, PARP1 dissociates from these promoters, thereby facilitating osteoclast differentiation. Previous studies have implicated caspase-1 in NLRP3 inflammasome-driven PARP1 degradation, which modulates NFATc1 activity during osteoclastogenesis [6, 20]. Here, we confirm the inhibitory role of PARP1 in osteoclast differentiation and present new evidence that caspase-11 directly mediates its cleavage. In vitro enzyme assays using recombinant caspase-11 demonstrated direct PARP1 cleavage. Furthermore, Western blot analysis using an antibody that recognizes cleaved PARP1 (Asp214) revealed a cleavage mechanism analogous to that of apoptosis-related caspases, such as caspase-3 and -7 [30].

Interestingly, cleaved PARP1 (p89 fragment) was detected in the cytoplasmic fraction of RANKL-treated BMMs and RAW 264.7 cells. However, subcellular fractionation experiments revealed that full-length PARP1 is exclusively present in the nuclear compartment, regardless of RANKL stimulation. These results suggest that the cytoplasmic p89 fragment arises from nuclear cleavage and subsequent redistribution rather than de novo cleavage within the cytoplasm. This interpretation aligns with the well-established nuclear localization of PARP1 and the caspase-mediated cleavage patterns observed during differentiation and apoptosis [31]. Nonetheless, recent studies have reported functional roles for cytoplasmic PARP1 under specific stress-related conditions, such as viral infection or LPS-induced microglial activation [32, 33]. While these findings highlight the potential for context-dependent translocation and activity of PARP1, our results suggest that cytoplasmic cleavage is unlikely to play a significant role in RANKL-induced osteoclastogenesis.

The role of caspase-11 in osteoclastogenesis was further validated using caspase-11 knockout BMMs. RANKL-induced PARP1 cleavage was significantly reduced in knockout cells compared to wild-type cells. Additionally, treatment with the PARP1 inhibitor rucaparib restored osteoclast differentiation in caspase-11-deficient BMMs, confirming that caspase-11 modulates osteoclastogenesis via PARP1. However, rucaparib treatment did not fully restore osteoclast differentiation to wild-type levels, suggesting that caspase-11 may influence osteoclastogenesis through additional substrates or pathways besides PARP1. Further studies are needed to identify these potential targets and fully elucidate the broader role of caspase-11 in osteoclast biology.

Although caspase-11, like most caspases, lacks a nuclear localization signal motif, our study provides novel evidence that it translocates to the nucleus upon RANKL stimulation. Western blot analysis confirmed the presence of both pro- and mature forms of caspase-11 in the nucleus following RANKL treatment. However, whether caspase-11 undergoes maturation in the cytoplasm before nuclear import or is directly processed within the nucleus remains unclear. Identifying RANKL-responsive substrate-like proteins that facilitate as cytoplasmic-to-nuclear shuttling carriers for caspase-11 is an important area for further investigation [34].

In vivo studies revealed that adult caspase-11 knockout mice exhibit greater bone volume than wild-type mice, likely due to reduced osteoclast number and activity. In vitro studies using BMMs further confirmed that caspase-11 deficiency impairs osteoclast differentiation and activity, consistent with increase bone mass in knockout mice. Interestingly, even young caspase-11 knockout mice (4 weeks old) displayed increased bone mass, a developmental stage where bone formation dominates over resorption [35]. This finding suggests that caspase-11 may regulate both bone resorption and formation, a hypothesis warranting further exploration. A key limitation of this study is the use of conventional knockout mice, which do not allow for cell type-specific analysis of caspase-11 function. While in vitro experiments with caspase-11-deficient BMMs clarified its role in osteoclastogenesis, bone remodeling involves multiple cell types, including osteoblasts, osteoclasts, and chondrocytes. Future studies employing conditional knockout models will be essential to delineate the cell-specific roles of caspase-11 in bone remodeling and provide a more physiologically relevant understanding of its function.

Concerns regarding the long-term use of current osteoporosis treatments, such as bisphosphonates and RANKL inhibitors, stem from potential adverse effects, including atypical fractures and osteonecrosis of the jaw [36, 37]. This underscores the need for novel therapeutic strategies targeting osteoclast-specific pathways. Our previous study demonstrated that the NLRP3 inflammasome contributes to age-related alveolar bone loss through both inflammation-dependent and -independent mechanisms, and that the NLRP3 inhibitor MCC950 effectively prevents this condition [19]. Similarly, inflammasome-targeted approaches are being explored for treating alveolar bone loss [38]. Given their role in inflammasome activation, inflammatory caspases are emerging as promising drug targets, with several inhibitors currently under development [39]. While pyroptosis inhibitors have been proposed for caspase-11-mediated diseases, they predominantly target GSDMD rather than caspase-11 itself [40]. The absence of selective caspase-11 inhibitors may be due to its limited substrate specificity compared to caspase-1 [41]. Nevertheless, elucidating the role of caspase-11 in osteoporosis may facilitate the development of selective inhibitors with therapeutic potential.

To explore the feasibility of targeting caspase-11 in osteoclast-mediated bone loss, we evaluated the dual caspase-1/-11 inhibitor VX-765. VX-765 is an orally bioavailable prodrug metabolized to VRT-043198, which potently inhibits human caspase-1 (Ki: 0.8 nM) and caspase-4 (Ki: <0.6 nM) in vitro [21]. Given that mouse caspase-11 is the functional ortholog of human caspase-4, its inhibition VX-765 is inferred based on sequence and functional homology. Originally developed for caspase-1-mediated inflammatory diseases involving IL-1β [42, 43], VX-765 demonstrated efficacy in inhibiting osteoclastogenesis in vitro and significantly attenuated OVX-induced bone loss by approximately 65% in vivo. This bone-protective effect correlated with reduced osteoclast numbers and decreased expression of osteoclast-specific markers. Notably, although OVX-induced bone loss was associated with increased caspase-11 activation, VX-765 treatment did not diminish the levels of its mature subunits (p38/p30), consistent with previous findings that VX-765, as a reversible inhibitor, does not prevent proteolytic processing of pro-caspase-1 [42].

The relative contributions of caspase-1 and caspase-11 to the anti-resorptive effects of VX-765 remain to be clarified. OVX-induced estrogen deficiency activates the NLRP3–caspase-1–IL-1β axis [44], suggesting that VX-765’s action may partly result from inhibiting caspase-1 and subsequently reducing systemic inflammation. In contrast, inhibition of caspase-11 may directly impair osteoclast differentiation and activity. These findings highlight the translational potential of targeting human caspase-4, the functional equivalent of murine caspase-11, as a therapeutic approach for osteolytic bone diseases. Nevertheless, the development of selective inhibitors or conditional knockout models is essential to understanding the specific role of caspase-11 in bone homeostasis.

In summary, this study identifies caspase-11 as a novel regulator of osteoclast differentiation through a unique mechanism involving the proteolytic degradation of PARP1, a transcriptional repressor of osteoclastogenesis. These findings offer new insights into the role of caspase-11 in bone metabolism and emphasize its potential as a therapeutic target for osteolytic diseases.

## Materials and methods

### Mice

C57BL/6 J mice were obtained from Damool Science (Daejeon, Korea). The Jackson Laboratory (Bar Harbor, ME) supplied the caspase-11 knockout mice (*Casp4*^*tm1Yuan*^, #024698) [45]. The genotypes of the mice were confirmed by semi-quantitative polymerase chain reaction (PCR) using primers from the indicated supplier (Supplementary Table 1). This study included both male and female animals to ensure the generalizability of the findings across sexes. Male mice were primarily used to minimize the influence of hormonal fluctuations, particularly estrogen, on bone remodeling and RANKL-induced osteoclastogenesis. Conversely, female mice were exclusively utilized for the OVX-induced bone loss model to study the impact of estrogen deficiency on bone resorption. Unless otherwise stated, key in vivo and in vitro experiments yielded consistent findings across each sex. No animals were excluded from the analysis. All mice were maintained in accordance with the guidelines of the Institutional Animal Care and Use Committee of Chonnam National University (CNU IACUC-YB-2024-84). This study also followed the ARRIVE guidelines for preclinical studies.

### Cell cultures

Bone marrow cells were isolated from the long bones of 6- to 8-week-old C57BL/6 wild-type or caspase-11 knockout mice. To generate BMMs, the isolated bone marrow cells were cultured for 4 days in α-MEM (Gibco, Grand Island, NY) supplemented with 10% fetal bovine serum (FBS; Gibco), 100 U/mL penicillin−streptomycin (Gibco), and recombinant mouse M-CSF (30 ng/mL; Biolegend, San Diego, CA). Mouse monocyte/macrophage RAW 264.7 cells (Korean Cell Line Bank, Seoul, Korea) were maintained in α-MEM containing 10% FBS and antibiotics and authenticated based on morphology and growth behavior.

To induce osteoclast differentiation, BMMs were seeded at a density of 3 × 10^5^ cells/mL and cultured for 3 to 4 days in a medium containing M-CSF (30 ng/mL) and recombinant mouse RANKL (100 ng/mL; Peprotech, Rocky Hill, NJ). In a parallel experiment, RAW 264.7 cells were seeded at a 1.5 × 10^5^ cells/mL density and cultured with 100 ng/mL RANKL. During this period, the cells were treated with Ac-LEVD-CHO (caspase-11 inhibitor; Sigma, St. Louis, MO), BAY 11-7082 (NF-κB inhibitor; Sigma), LDC7559 (GSDMD/pyroptosis inhibitor, Selleckchem, Houston, TX) or VX-765 (caspase-1/-11 inhibitor; AdooQ, Irvine, CA), as indicated. The medium was replaced daily. Osteoclast formation was assessed by tartrate-resistant acid phosphatase (TRAP) staining.

### Inflammasome activation

For canonical inflammasome activation, BMMs (5 × 10^5^ cells/mL) were primed with 100 ng/mL *E. coli* LPS (Sigma) for 6 h in a complete medium, followed by replacement with Opti-MEM (Gibco). The cells were then stimulated with 3 mM ATP (Sigma) for 30 minutes. The culture supernatant and cell lysate were subsequently collected for further analyses. For non-canonical inflammasome activation, BMMs (5 × 10^5^ cells/mL) were primed with 100 ng/mL LPS for 16 h. The medium was then replaced with Opti-MEM and cells were transfected with LPS (final concentration of 25 μg/mL) for 8 h using FuGENE HD transfection reagent (final concentration of 0.6% v/v; Promega, Madison, WI). Culture supernatants were concentrated by methanol/chloroform protein precipitation. Inflammasome activation was assessed using Western blot analysis of supernatants and cell lysates.

### Micro-computed tomography (μ-CT) analysis

Formalin-fixed bone specimens were scanned using a Skyscan 1172 X-ray microtomography system (Bruker, Kontich, Belgium) with an isotropic voxel size of 15 μm and an X-ray voltage of 50 kV and current of 200 μA. The acquisition of three-dimensional (3D) images was facilitated using Skyscan NRecon software, followed by CT-analyzer (CTan) software analysis. The 3D rendering of bone structures was accomplished using Mimics software (version 14.0, Materialise). Although group allocation was not concealed, two investigators (XP and JWK) performed the image processing and quantitative analyses independently to minimize subjective bias. For the femur, trabecular bone volume was quantified within a volume of interest (VOI) located 0.54 mm proximal to the distal epiphyseal growth plate, extending a height of 1 mm. Cortical bone volume was measured at the mid-diaphysis over a length of 0.5 mm. Vertebral bone volume was assessed at the middle (50%) and central (45%) regions of the fifth lumbar vertebra (L5). The integrity of the alveolar bone was assessed by measuring the cementoenamel junction to the alveolar bone crest (CEJ-ABC) distance on the buccal side of the mandible. Specifically, the measurement was taken at the distal root of the first molar, both roots of the second molar, and the root of the third molar. Additionally, bone volume was measured at the interproximal regions between the first and second molars and the second and third molars.

Additional experimental details are provided in the Supplementary Materials and Methods section of the Supplementary Information. Full-length uncropped Western blot images are available in the file labeled “Original Western blots”.

## Supplementary information

Supplementary information

Original Western blots

## Data Availability

All data supporting the findings of this study are available from the corresponding author upon reasonable request.
