# Supplementary material for: Non-pyroptotic caspase-11 activity regulates osteoclastogenesis and pathological bone loss
Source: Cell Death Differ. 2025 Oct 22;33(4):717–31. doi: 10.1038/s41418-025-01596-3 (PMC13076654; doi:10.1038/s41418-025-01596-3)
Supplement: Supplementary file 1 — Supplementary information [file 41418_2025_1596_MOESM1_ESM.docx]

**Supplementary Information**

**Non-pyroptotic caspase-11 activity regulates osteoclastogenesis and pathological bone loss**

Xianyu Piao^1,2,*^, Ju Han Song^1,2,*^, Jung-Woo Kim^1,2^, Seung-Hee Kwon^1,2^, Sin-Hye Oh^1,2^, Sangita Sharma^1,2^, Suk-Gyun Park^1,2^, Zhao Wang^1,2^, Zhiyu Fang^1,2^, Je-Hwang Ryu^1,2^, Nacksung Kim^2,3^, and Jeong-Tae Koh^1,2^

^1^*Department of Pharmacology and Dental Therapeutics, School of Dentistry, Chonnam National University, Gwangju, Republic of Korea*

^2^*Hard-tissue Biointerface Research Center, School of Dentistry, Chonnam National University, Gwangju, Republic of Korea*

*^3^Department of Pharmacology, Chonnam National University Medical School, Gwangju, Republic of Korea*

**Corresponding Author**: Dr. Jeong-Tae Koh, Department of Pharmacology and Dental Therapeutics, School of Dentistry, Chonnam National University, 77 Yongbong-ro, Buk-gu, Gwangju 61186, Republic of Korea; e-mail: jtkoh@chonnam.ac.kr; phone: +82-62-530-4861

***Authorship Note**: XP and JHS contributed equally to this study.

**Supplementary Materials and Methods**

**TRAP staining and pit formation assay**

To evaluate osteoclast formation, cells cultured in 96-well plates under the osteoclastogenic medium were fixed with formalin for 15 minutes and then stained with a TRAP staining kit (Cosmo Bio, Tokyo, Japan) according to the manufacturer’s protocol. The resulting staining images were then captured using a Lionheart FX Cell Imager (BioTek, Winooski, VT). The number of osteoclasts was determined by counting TRAP-positive multinucleated cells with three or more nuclei per well.

To assess bone resorption activity, BMMs were seeded on a 48-well plate coated with calcium phosphate from a bone resorption assay kit (Cosmo Bio) and exposed to RANKL (100 ng/mL) in the presence or absence of other stimuli. After 7 days, the cells were removed using phosphate-buffered saline (PBS) containing 2% Triton X-100. Using a digital camera, pit formation images were obtained from at least four representative areas per well under the DM IL light microscope (Leica, Wetzlar, Germany) and analyzed using ImageJ software (NIH, Bethesda, MD). Results are expressed as a percentage of the pit area of the total area.

**Enzyme-linked immunosorbent assay (ELISA)**

IL-1β secretion was quantified in culture supernatants using the ELISA MAX Deluxe mouse IL-1β kit (BioLegend), according to the manufacturer’s instructions. Supernatants were collected from BMMs under stimulated with RANKL or under the conditions described in the Inflammasome activation section. Absorbance was measured at 450 nm using a Multiskan GO microplate reader (Thermo Fisher Scientific, Vantaa, Finland).

**LDH release assay**

To evaluate pyroptotic cell death, LDH activity was measured in the same culture supernatants used for ELISA, using the EZ-LDH assay kit (DoGenBio, Seoul, Korea). Absorbance was measured at 450 nm using a Multiskan GO microplate reader. The cell death rate was calculated as the percentage of LDH release compared to the maximum LDH release (100%) obtained by lysing cells with hydrogen peroxide.

**Small interfering RNA (siRNA) transfection**

For transient gene knockdown experiments, BMMs were transfected with siRNAs using Lipofectamine 2000 reagent (Invitrogen, Carlsbad, CA) for 6 hours according to the manufacturer’s instructions and then incubated in fresh media for an additional 24 hours. Pre-designed and validated siRNAs were utilized to target *caspase-11* and *poly(ADP-ribose) polymerase 1* (*Parp1*). The sequences used were as follows: *caspase-11*: 5’−ugaagacuuaggcuacgautt−3’ and 5’−aucguagccuaagucuucatt−3’; *Parp1*: 5’−cugacauuaaggugguugatt−3’ and 5’−ucaaccaccuuaaugucagtt−3’. A scrambled siRNA was employed as the negative control. All siRNAs were obtained from Bioneer (Daejeon, Korea).

**qRT-PCR analysis**

Total RNA was extracted using TRIzol reagent (Ambion, Carlsbad, CA) and converted to cDNA using M-MLV reverse transcriptase (Promega). qRT-PCR was performed using SYBR Green PCR Master Mix (Applied Biosystems, Valencia, CA) with specific primers on a QuantStudio 6 Flex Real-Time PCR System (Applied Biosystems). Data were analyzed using the 2^-ΔΔCt^ method and normalized to the *18S rRNA* gene. A list of primers used is delineated in S Table 1.

**Western blot analysis**

Whole-cell lysates were extracted using Mammalian Cell Lysis Buffer (CST, Danvers, MA) containing a protease inhibitor cocktail (GenDepot, Baker, TX). Cytoplasmic and nuclear cell fractions were extracted using the NE-PER nuclear and cytoplasmic extraction reagent kit (Thermo Fisher Scientific). Protein concentrations were determined using the DC protein assay kit (Bio-Rad, Hercules, CA). Proteins were separated on a 10−15% SDS-PAGE gel and transferred to a PVDF membrane (Cytiva, Germany). The membranes were then blocked in Tris-buffered saline (TBS) buffer containing 0.1% Tween-20 and 5% nonfat milk for 30 minutes. Thereafter, the membranes were incubated with primary antibodies at 4℃ overnight, followed by incubation with HRP-conjugated secondary antibodies for 1 hour. The membranes were washed at least thrice between each step using TBS buffer. Immunoblots were detected using an ECL solution (Millipore, Burlington, MA) and Western blot imaging systems (Azure Biosystems, Dublin, CA). The antibodies used are listed in S Table 2.

**Immunofluorescence staining**

BMMs and RAW 264.7 cells were seeded on 8-well Cell Culture DLux slides (SPL Life Sciences) and treated with RANKL for 24 hours. Following fixation with 4% paraformaldehyde and permeabilization with 0.1% Triton X-100, cells were incubated with a rat anti-caspase-11 antibody (Novus, Centennial, CO) at 4℃ overnight. After washes, cells were incubated with Alexa-Fluor 488-conjugated anti-rat IgG (H+L) at room temperature for 1 hour. The nucleus was stained with 4’,6-diamidino-2-phenylindole (DAPI, 10 μg/mL). The slides were then mounted in an aqueous mounting medium. Fluorescence images were captured using a Zeiss AXIO Scope.A1 fluorescence microscope (Jena, Germany). The subcellular localization of caspase-11 was analyzed using the RGB profile calculated using the ImageJ plugin.

**Colorimetric assay for caspase-11 activity**

Caspase-11 activity was determined using the caspase-4 colorimetric assay kit (BioVision, Milpitas, CA), according to the manufacturer’s instructions. Briefly, 3 × 10^6^ BMMs were seeded in a 10 cm culture dish and treated with RANKL. Cells were harvested at various times and lysed with caspase assay buffer. In total, 200 μg protein was used per reaction to determine caspase-11 activity using the substrate Ac-LEVD-pNA. Absorbance was measured at 405 nm using a Multiskan GO microplate reader. Data are expressed as a fold change relative to the unstimulated control.

**Flow cytometric analysis**

BMMs from caspase-11 wild-type and knockout mice were incubated with PE-conjugated anti-F4/80 and APC-conjugated anti-RANK antibodies in binding buffer (PBS containing 0.5% FBS and 0.1% sodium azide) at room temperature for 20 minutes. Following this incubation, the cells were washed with binding buffer and immediately analyzed using an Accuri C6 Plus flow cytometer (BD Biosciences, San Jose, CA). Analysis was performed on F4/80-positive cells to determine RANK expression on the cell surface. Isotype control antibodies were utilized as negative controls, and antibody details are listed in S Table 2.

**Caspase-11 overexpression constructs and transduction**

Full-length caspase-11 cDNA was amplified by PCR using primers containing *BamHI* and *XhoI* restriction sites (forward: 5'−cccggatccatggctgaaaacaaacac−3'; reverse: 5'−aaactcgagtcagttgccaggaaagag−3'). The PCR product was purified, digested with *BamHI* and *XhoI*, and cloned into the pMX retroviral vector to generate the full-length expression construct, pMX-C11. To construct a nuclear-targeted, active form of caspase-11, we engineered a retroviral vector (pMX-NLS-aC11) that encodes the large and small catalytic domains of caspase-11 fused to a nuclear localization signal (NLS) derived from simian virus 40 (SV40). This was achieved by PCR amplification from the pMX-C11 backbone using the following primers: forward, 5’−ccggatccatgccaaagaagaagagaaaggttccaggcagccaccatggtgaa−3’; reverse, 5’−aaactcgagtcagttgccaggaaagag−3’. The recombinant constructs were transfected into Plat-E packaging cells using Lipofectamine 2000 according to the manufacturer’s protocol. Viral supernatants were collected after 48 hours, filtered through a 0.2 μm syringe filter, and used to infect BMMs from caspase-11 knockout mice. The BMMs were infected by incubating them with a 30-fold diluted viral supernatant supplemented with 3 μg/mL polybrene (Sigma) for 4 hours. This was followed by replacing the media and incubating the cells for an additional 24 hours incubation. The cells were then treated with RANKL. Viral supernatant from Plat-E cells transfected with and empty pMX vector (pMX-Ctrl) was used as the control.

**In vitro enzyme assay**

Whole-cell lysates (20 μg/reaction) extracted from naive BMMs using caspase assay buffer (Biovision) or recombinant mouse full-length PARP1 (50 ng/reaction; Sino Biological) were incubated with recombinant mouse caspase-11 (10 U/reaction; Enzo Life Sciences) in assay buffer containing 100 mM dithiothreitol, with or without Ac-LEVD-CHO (caspase-11 inhibitor, 200 μM) at 37℃ for 30−60 minutes. The reactions were stopped by mixing with 5× loading buffer and boiling at 95℃. The samples were subsequently analyzed using Western blotting.

**Animal models for RANKL-related bone loss**

To establish an age-related bone loss model, male C57BL/6 mice were fed a chow diet and allowed to age spontaneously. Femurs and tibias were harvested from mice aged 6 months and over 24 months. Femurs were used for histomorphometric analysis, while tibias were used for protein analysis.

To establish an ovariectomy (OVX)-induced bone loss model, 12-week-old female C57BL/6 mice were anesthetized, and a midline incision was made in the abdominal skin. The ovaries on both sides were removed and the ends were closed with sutures. Sham-operated mice underwent the same procedure without ovary removal. Both groups received gentamicin (50 mg/kg) to prevent infection during the one-week recovery period. The mice were euthanized eight weeks after surgery, and their femurs were harvested for analysis.

For the periodontitis-induced alveolar bone loss model, 10-week-old male C57BL/6 mice were anesthetized, and a 5-0 ligature was placed around the maxillary second molar neck. Mice were euthanized on the fourth day after ligation, and their maxillary regions were carefully excised. Either fixing in formalin or storing at -80℃ was employed to preserve alveolar bone samples for further analysis.

**RANKL injection for inducing bone loss**

Twelve-week-old male C57BL/6 wild-type and caspase-11 knockout mice were randomly divided into two groups per strain using an alternating order of cage assignment to ensure unbiased group allocation. One group received intraperitoneal injections of recombinant RANKL protein (1 mg/kg), while the other received an equal volume of the vehicle as a control. Injections were administered on the first and second days. The femur and vertebral bones were collected on the fourth day and immediately processed for further analysis, including bone mass measurement, osteoclast counting, and gene expression profiling.

**Effect of VX-765 on OVX-induced bone loss model**

Ten-week-old female C57BL/6 mice underwent bilateral OVX to establish an osteoporosis model, while sham-operated mice served as controls. After a one-week recovery period, the OVX and sham-operated mice were randomly divided into vehicle- and VX-765 (100 mg/kg)-treated groups using alternating order based on cage assignment. The VX-765 stock solution (100 mg/mL in DMSO) was stored at -20℃. Before each administration, 20 μL of the VX-765 stock solution was progressively mixed with 20 μL polyethylene glycol 400 (PEG400; Sigma), 5 μL Tween-80 (Sigma), and 55 μL PBS, yielding a final injection volume per mouse of 100 μL. An equivalent volume of the vehicle was prepared using the aforementioned solvents, excluding VX-765. Intraperitoneal injections were administered thrice weekly for four weeks. During the treatment period, body weights were measured weekly to monitor health and potential drug toxicity. At the end of the four-week treatment period, femurs were harvested for further analysis to evaluate the effects of VX-765 on osteoclast-mediated bone loss.

**Histochemical analysis**

Formalin-fixed bone samples were decalcified with 0.5 M EDTA (pH 7.4) for 5 days, then dehydrated, embedded in paraffin, and sectioned into 5 μm thick slices. After deparaffinization and rehydration, sections were stained with H&E to evaluate general tissue morphology. To detect osteoclasts, tissue sections were stained with a TRAP staining kit and counterstained with methyl green. Images were acquired using an Aperio CS2 slide scanner (Leica Biosystems). Although group allocation was not blinded, two investigators (JHS and SHK) independently quantified TRAP-positive osteoclasts to reduce observer bias. TRAP-positive cells were quantified in the primary spongiosa of the distal metaphysis and along the endosteal surface of the diaphysis.

**Statistical analysis**

Sample sizes were determined based on previous studies in similar experimental settings. For in vivo studies, no statistical method was used to predetermine the sample size but a minimum of 5–10 mice per group was used, as commonly adopted in bone loss models. The sample size for each group (n) is specified in the corresponding figure legend. All in vitro experiments were performed independently at least twice with consistent results. The figures presented show representative data from one experiment, with values expressed as the mean ± standard deviation (SD) of triplicate technical replicates. Statistical significance was determined by an unpaired Student’s *t*-test or a one-way analysis of variance (ANOVA) followed by a Tukey’s post hoc test (GraphPad Prism, version 8.0). A *p*-value less than 0.05 was considered statistically significant.

**Supplementary Tables**

**S Table 1** List of the primers used in this study

| Gene | Sequence (5’ → 3’) | | Application |
| --- | --- | --- | --- |
| *Trap* | F : | tcc gtg ctc ggc gat gga cca ga | qRT-PCR |
|  | R : | ctg gag tgc acg atg cca gcg aca |  |
| *Caspase-11* | F : | ccg aga caa aac agg agg ct | qRT-PCR |
|  | R : | aag gtt gcc cga tca atg gt |  |
| *c-Fos* | F : | ggg aat ggt gaa gac cgt gt | qRT-PCR |
|  | R : | gca atc tca gtc tgc aac gc |  |
| *Ctsk* | F : | tac cca tat gtg ggc cag ga | qRT-PCR |
|  | R : | ata gcc cac cac caa cac tg |  |
| *Mmp-9* | F : | tgg tct tcc cca aag acc tg | qRT-PCR |
|  | R : | agg ttt gga atc gac cca cg |  |
| *Nfatc1* | F : | ctc gaa aga cag cac tgg agc at | qRT-PCR |
|  | R : | cgg ctg cct tcc gtc tca ta |  |
| *Tnfrsf11a* | F : | gca tcc ctt gca gct caa ca | qRT-PCR |
|  | R : | atg gaa gag ctg cag acc ac |  |
| *18s rRNA* | F : | ggc cgt tct tag ttg gtg ga | qRT-PCR |
|  | R : | ccc gga cat cta agg gca tc |  |
| *Caspase-11* | F : | aca att gcc act gtc cag gt | Genotyping |
|  | R (Mt) : | cgc ttc ctc gtg ctt tac ggt at |  |
|  | R (WT) : | cat tgc tga cct tat ttc tgt atg g |  |

Abbreviations: F, forward; R, reverse; qRT-PCR, quantitative real-time reverse transcription-polymerase chain reaction; Mt, mutant; WT, wild-type.

**S Table 2**. List of the antibodies used in this study.

| Antibody | Brand | Cat. No | Application | Dilution |
| --- | --- | --- | --- | --- |
| β-Actin | SCBT | sc-47778 | WB | 1:2,000 |
| Caspase-1 | CST | 2225 | WB | 1:1,000 |
| Caspase-11 | Abcam | ab180673 | WB | 1:1,000 |
|  |  |  | IP | 1:100 |
|  | Novus | NB120-10454 | WB | 1:1,000 |
|  |  |  | IF | 1:100 |
| CTSK | SCBT | sc-48353 | WB | 1:1,000 |
| c-Fos | CST | 2250 | WB | 1:1,000 |
| GSDMD | CST | 39754 | WB | 1:1,000 |
| IL-1β | CST | 12507 | WB | 1:1,000 |
| IκB | SCBT | sc-371 | WB | 1:1,000 |
| p-IκB (Ser32) | CST | 2859 | WB | 1:1,000 |
| Lamin B | SCBT | sc-56145 | WB | 1:1,000 |
| MEK2 | CST | 9125 | WB | 1:1,000 |
| NFATc1 | SCBT | sc-13033 | WB | 1:1,000 |
| p65 | CST | 6956 | WB | 1:1,000 |
| p-p65 (Ser536) | CST | 3033 | WB | 1:1,000 |
| PAR | R&D | 4335-MC-100 | WB | 1:1,000 |
| PARP1 | CST | 9542 | WB | 1:1,000 |
|  | Abcam | ab227244 | IF | 1:100 |
| Cleaved PARP1 (Asp214) | CST | 94885 | WB | 1:1,000 |
| F4/80-PE | Biolegend | 123109 | FC | 1:200 |
| RANK-APC | Biolegend | 119807 | FC | 1:200 |
| anti-Rabbit IgG-HRP | Invitrogen | A16096 | WB (2^nd^) | 1:10,000 |
| anti-Mouse IgG-HRP | Invitrogen | 31430 | WB (2^nd^) | 1:10,000 |
| anti-Rat IgG-HRP | Biolegend | 405405 | WB (2^nd^) | 1:15,000 |
| Rat IgG2a, k-PE | Biolegend | 400507 | FC (isotype) | 1:200 |
| Rat IgG2a, k-APC | Biolegend | 400511 | FC (isotype) | 1:200 |
| Rabbit IgG | Sigma | I8140 | IP (isotype) | 1:100 |
| anti-Rat IgG-A488 | CST | 4416 | IF (2^nd^) | 1:1,000 |

Abbreviations: A488, Alexa Fluor 488; APC, allophycocyanin; CST, Cell Signaling Technology; FC, flow cytometry; HRP, horseradish peroxidase; IF, immunofluorescence; IP, immunoprecipitation; WB, Western blot; PE, phycoerythrin

**Supplementary Figures**

**S Fig. 1**

**S Fig. 1 RANKL concentration-dependent increase in caspase-11 expression.**

Bone marrow-derived macrophages (BMMs) were treated with varying concentrations of RANKL for 6 hours. (**a**) qRT-PCR analysis of *caspase-11* and *c-Fos* mRNA levels. Data are expressed as the mean ± SD (n = 3). (**b**) Western blot analysis of caspase-11 expression. β-actin was used as a loading control, and NF-κB p65 served as a positive marker for RANKL signaling.

**S Fig. 2**

**S Fig. 2 Determination of the effective concentration of LDC7559 for inhibiting GSDMD pore formation.**

Bone marrow-derived macrophages (BMMs) were primed with 100 ng/ml LPS for 6 hours in the presence of the indicated concentration of LDC7559 (LDC), then stimulated with 5 mM ATP for 30 minutes. Western blotting was performed to evaluate both the pro form (Pro) and the cleaved N-terminal fragment (NT) of GSDMD in the culture supernatant (Sup) and the whole cell lysate (Lys). β-actin was used as a loading control for lysates.

**S Fig. 3**

**S Fig. 3 Efficacy of caspase-11 inhibition by siRNA or peptide inhibitor.**

(**a**, **b**) Bone marrow-derived macrophages (BMMs) transfected with siRNA targeting *caspase-11* (si-*C11*) were analyzed for caspase-11 mRNA levels by qRT-PCR (**a**) and protein levels by Western blotting (**b**). (**c**, **d**) BMMs were cultured with RANKL in the presence or absence of the caspase-11 inhibitor z-LEVD-CHO (C11*inhi*) for 12 hours. (**c**) Western blot analysis of caspase-11 expression. β-actin was used as a loading control. (**d**) Caspase-11 enzymatic activity assay in whole cell extracts, expressed as fold change relative to untreated control. Data are expressed as the mean ± SD of three independent experiments.

**S Fig. 4**

**S Fig. 4 Attenuated bone loss after RANKL injection in caspase-11 knockout mice.**

(**a**) Representative 3D μ-CT images of L5 vertebrae from wild-type (WT) and *caspase-11* knockout (*Casp-11* KO) mice treated with either vehicle or RANKL. Sagittal, coronal, and transverse views of L5 vertebrae are shown. (**b**) Quantification of morphometric parameters for L5 vertebral trabecular bone, including bone volume/tissue volume (BV/TV), trabecular thickness (Tb.Th), trabecular number (Tb.N), bone mineral density (BMD), and trabecular separation (Tb.Sp). Data are presented as box-plots with median (horizontal line) and minimum/maximum values (whiskers). Vehicle-treated groups: n = 5; RANKL-treated groups: n = 6. (**c**) Representative 3D μ-CT images of femoral cortical bone from WT and *Casp-11* KO mice treated with vehicle or RANKL. (**d**) Quantification of femoral cortical bone parameters. Data are presented as box-plots as described in (**b**). Vehicle-treated groups: n = 5; RANKL-treated groups: n = 6. n.s, not significant.

**S Fig. 5**

**S Fig. 5 Increased bone mass observed in young caspase-11 knockout mice.**

(**a**) Body weights of 4-week-old wild-type (WT, n = 10) and *caspase-11* knockout (*Casp-11* KO, n = 10) mice. Data are expressed as the mean ± SD. (**b**) Growth plate (GP) thickness measured at five different points along the distal femur and averaged per each mouse (n = 4 per group). (**c**) Representative H&E-stained longitudinal sections of the distal femur from WT and *Casp-11* KO mice, highlighting the growth plate and trabecular bone regions. (**d**) Representative 3D μ-CT images of the trabecular bone of the distal femur. (**e**) μ-CT analysis of trabecular bone morphometric parameters, including bone volume/tissue volume (BV/TV), trabecular thickness (Tb.Th), trabecular number (Tb.N), bone mineral density (BMD), and trabecular separation (Tb.Sp). Box-plots show median (horizontal line) and minimum/maximum values (whiskers). n.s, not significant. Sample size: n = 10 per group.

**S Fig. 6**

**S Fig. 6 PARP1 is a negative regulator of osteoclast differentiation.**

(**a**–**c**) Bone marrow-derived macrophages (BMMs) were transfected with control siRNA (si-Ctrl) or *Parp1* siRNA (si-*Parp1*) and cultured with RANKL for 3 days. (**a**) qRT-PCR analysis of *Parp1* and osteoclast marker gene expression. Data are expressed as the mean ± SD (n = 3). (**b**) Western blot analysis of osteoclast marker proteins, including NFATc1 and cathepsin K, with β-actin as a loading control. (**c**) Representative images of TRAP staining and quantification of TRAP-positive osteoclasts. Scale bar, 500 μm. (**d**–**f**) BMMs were treated with RANKL for 3 days in the presence or absence of rucaparib (Ruca) at the indicated concentrations. (**d**) qRT-PCR analysis of osteoclast-related gene expression. Data are expressed as the mean ± SD (n = 3). (**e**) Western blot analysis for PAR, PARP1 and osteoclast marker proteins, with β-actin as an internal control. (**f**) Representative images of TRAP staining and quantification of TRAP-positive osteoclasts (mean ± SD). Scale bar, 500 μm.

**S Fig. 7**

**S Fig. 7 Nuclear-targeted caspase-11 enhances PARP1 cleavage by RANKL treatment.**

(**a**) Schematic representation of a caspase-11 construct that encodes the catalytic domains fused to a nuclear localization signal (NLS). (**b**, **c**) Bone marrow-derived macrophages (BMMs) from caspase-11 knockout (KO) mice were infected with retroviral particles containing the indicated constructs and treated with or without RANKL for 24 hours. Western blot analysis of whole-cell lysates (**b**) and subcellular fractions (**c**) was performed to assess the expression and nuclear localization of caspase-11, as well as PARP1 cleavage. β-actin, MEK2, and lamin B were used as loading controls for the total, cytosolic, and nuclear fractions, respectively.

**S Fig. 8**

**S Fig. 8 Caspase-11 translocates to the nucleus and is involves in PARP1 processing during osteoclast differentiation in RAW 264.7 macrophages.**

(**a**) Western blot analysis of PARP1, caspase-11, NFATc1, and cathepsin K protein levels in RAW 264.7 cells treated with RANKL for the indicated time. β-actin was used as an internal control. (**b**) Western blot analysis of the cytosolic and nuclear fractions of RAW 264.7 cells treated with RANKL for 24 hours. MEK2 and lamin B were used as loading controls for the cytosolic and nuclear fractions, respectively. (**c**, **d**) Immunofluorescence analysis of caspase-11 localization in RAW 264.7 cells treated with RANKL for 24 hours. (**c**) The cells were stained with an anti-caspase-11 antibody and an Alexa 488-conjugated secondary antibody (green). Cell nuclei were counterstained with DAPI (blue). Scale bars, 10 μm. (**d**) Fluorescence intensity profiles were generated using ImageJ along the white line. PRV (Pearson’s R value) indicates colocalization efficiency.

**S Fig. 9**

**S Fig. 9 In vivo effects of VX-765 on ovariectomy-induced bone loss.**

Sham-operated or ovariectomized (OVX) mice were treated with vehicle or VX-765. (**a**) qRT-PCR analysis of *caspase-11* and osteoclast-related gene expression levels in whole long bones. Data are expressed as fold change relative to sham-operated control group (mean ± SD, n = 4 per group). (**b**) Western blot analysis of caspase-11 protein levels in whole long bones, with β-actin used as an internal loading control.
