# Supplementary material for: Non-pyroptotic caspase-11 activity regulates osteoclastogenesis and pathological bone loss
Source: Cell Death Differ. 2025 Oct 22;33(4):717–31. doi: 10.1038/s41418-025-01596-3 (PMC13076654; doi:10.1038/s41418-025-01596-3)
Supplement: Supplementary file 2 — Original Western blots [file 41418_2025_1596_MOESM2_ESM.pdf]

Supplemental Material (original Western blots)

Fig. 1b

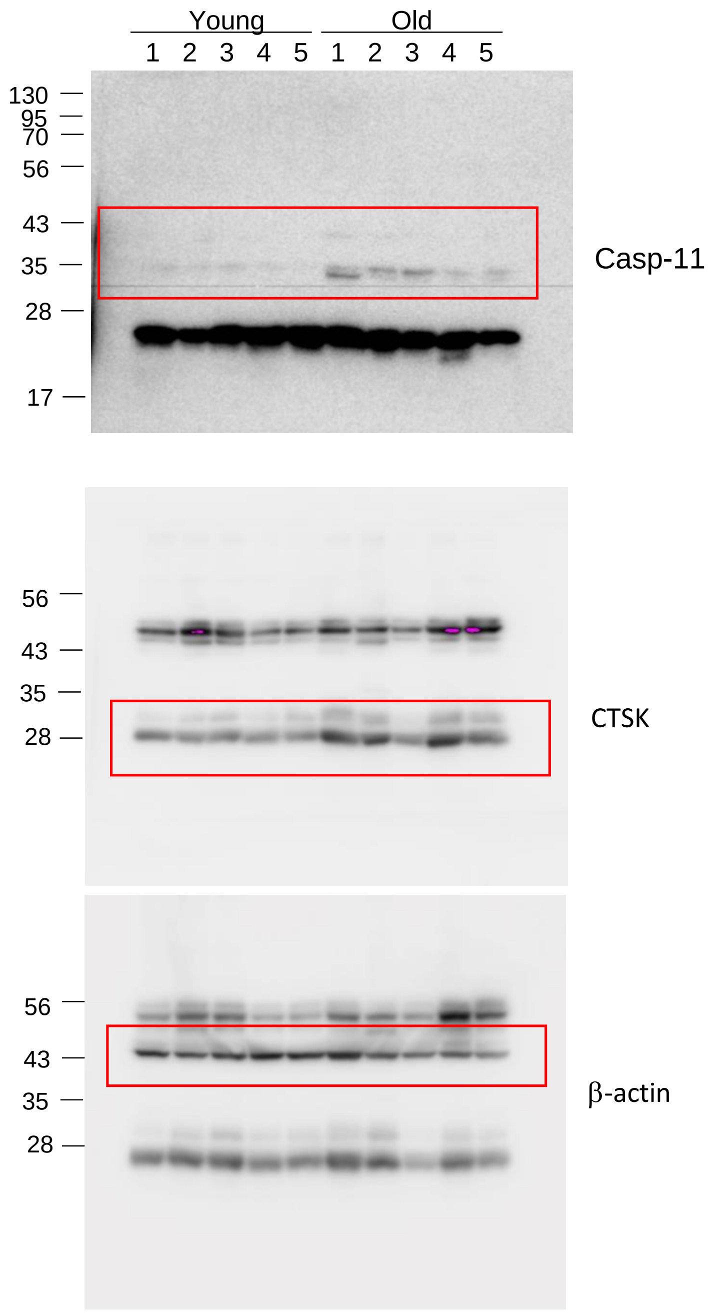

Fig. 1d

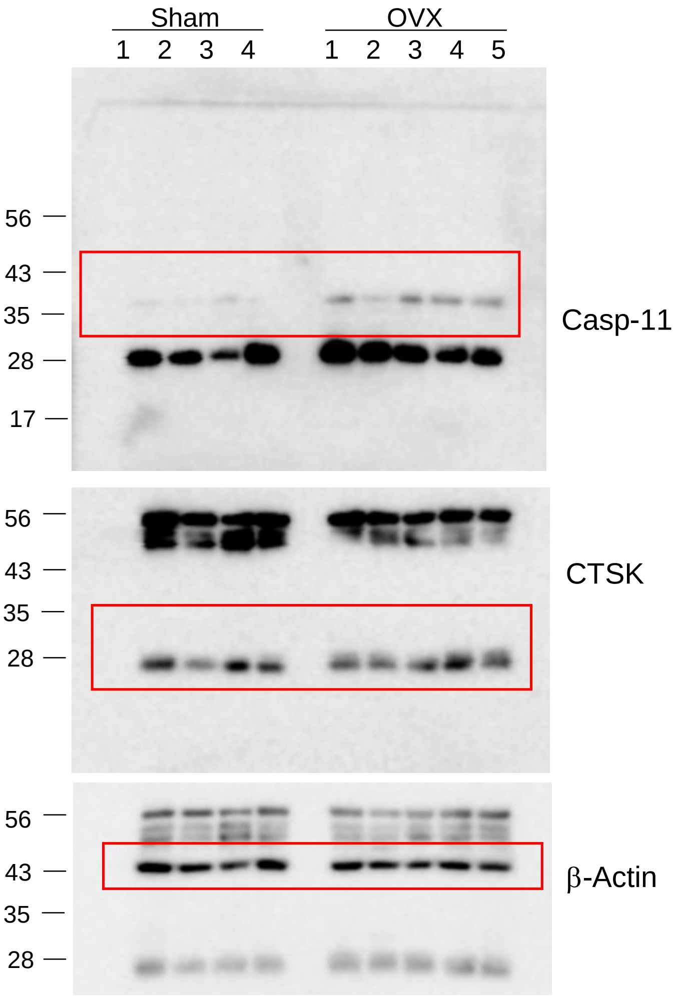

Sham
OVX
1 2 3 4
1 2 3 4 5
56
43
35
28
17
Casp-11
CTSK
β-Actin

Fig. 1f

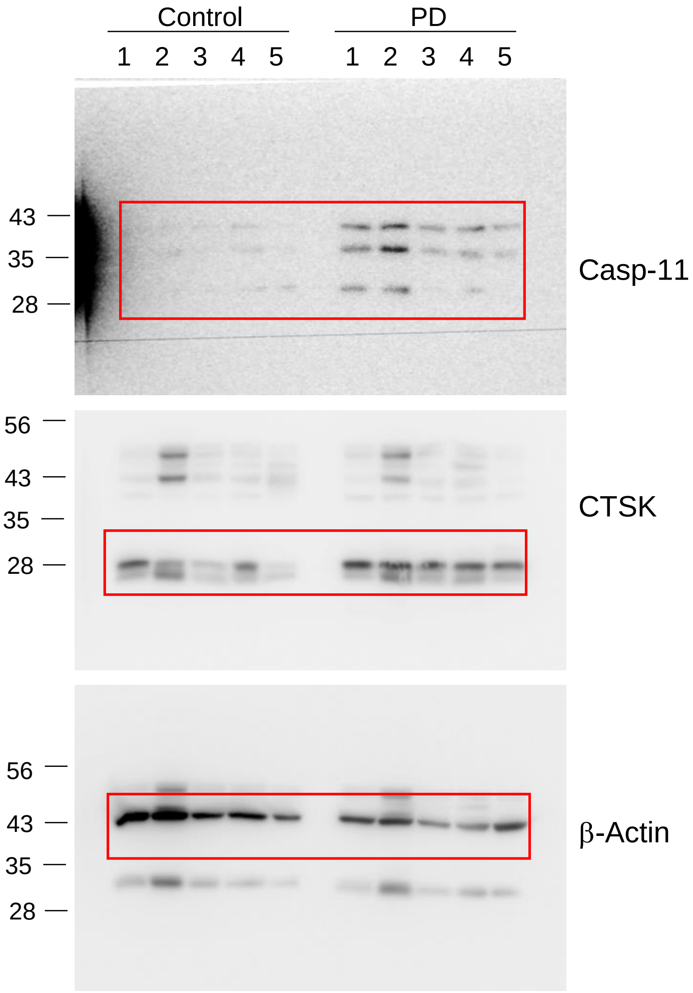

Control
PD
1 2 3 4 5
1 2 3 4 5
56
43
35
28
Casp-11
CTSK
β-Actin

Fig. 2b

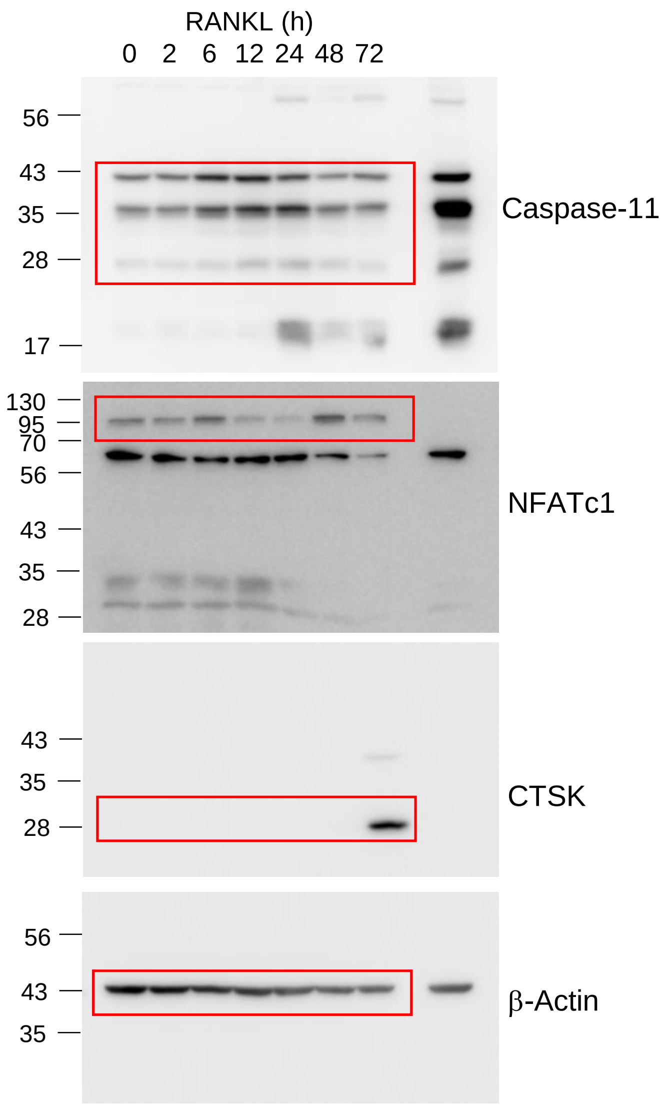
RANKL (h)
0 2 6 12 24 48 72
56
43
35
28
17
Caspase-11
130
95
70
56
43
35
28
NFATc1
43
35
28
CTSK
56
43
35
β-Actin

Fig. 2e

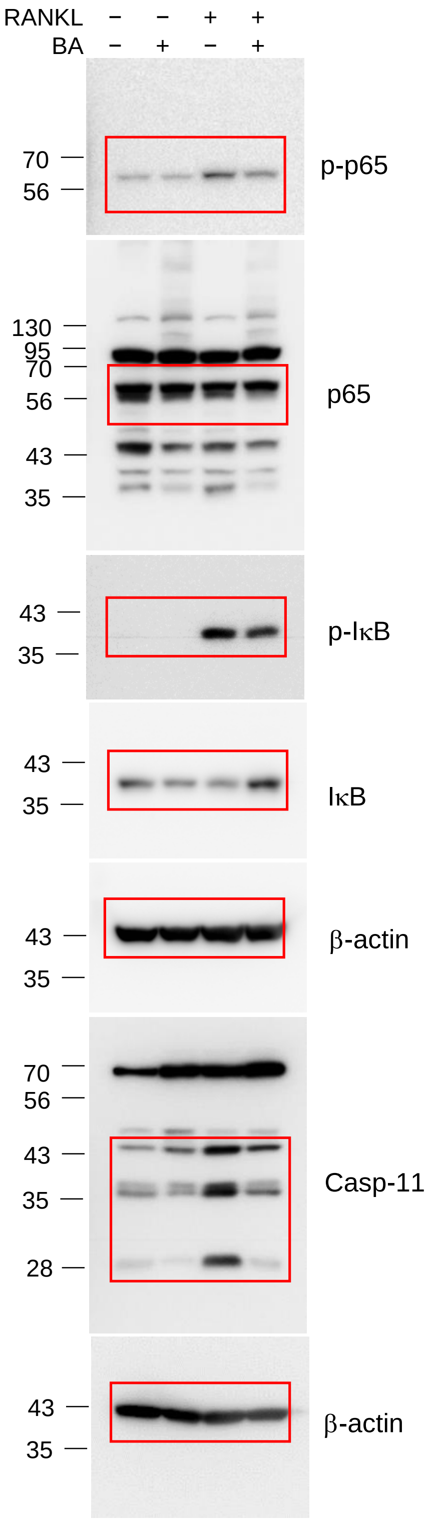
RANKL − − + +
BA − + − +
70
56
p-p65
130
95
70
56
43
35
p65
43
35
p-IκB
43
35
IκB
43
35
β-actin
70
56
43
35
28
Casp-11
43
35
β-actin

Fig. 2h

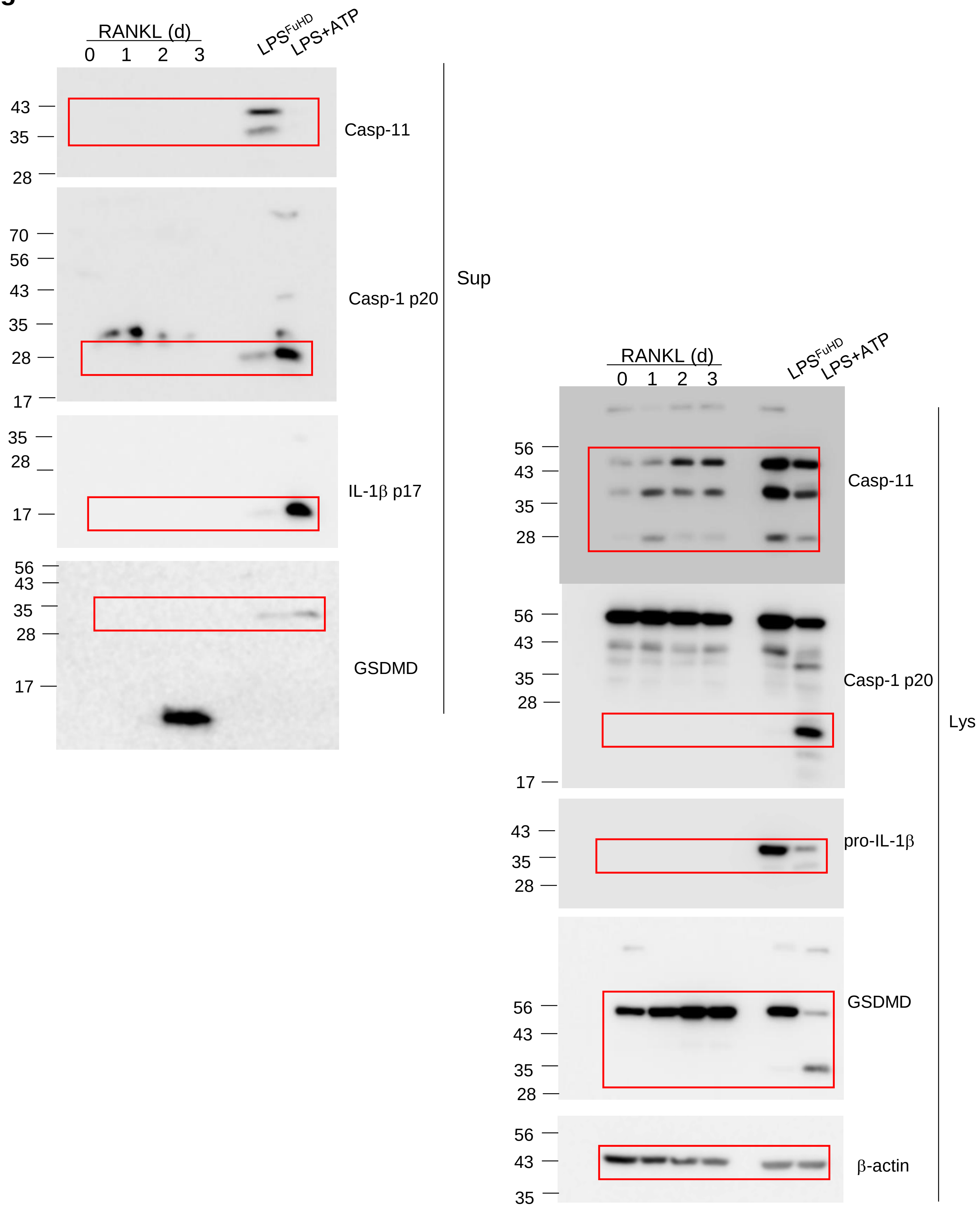
RANKL (d)
0 1 2 3
LPSFuHD
LPS+ATP
43
35
28
Casp-11
70
56
43
35
28
17
Casp-1 p20
35
28
17
IL-1β p17
56
43
35
28
17
GSDMD
Sup
RANKL (d)
0 1 2 3
LPSFuHD
LPS+ATP
56
43
35
28
Casp-11
56
43
35
28
17
Casp-1 p20
43
35
28
pro-IL-1β
56
43
35
28
GSDMD
56
43
35
β-actin
Lys

Fig. 2j

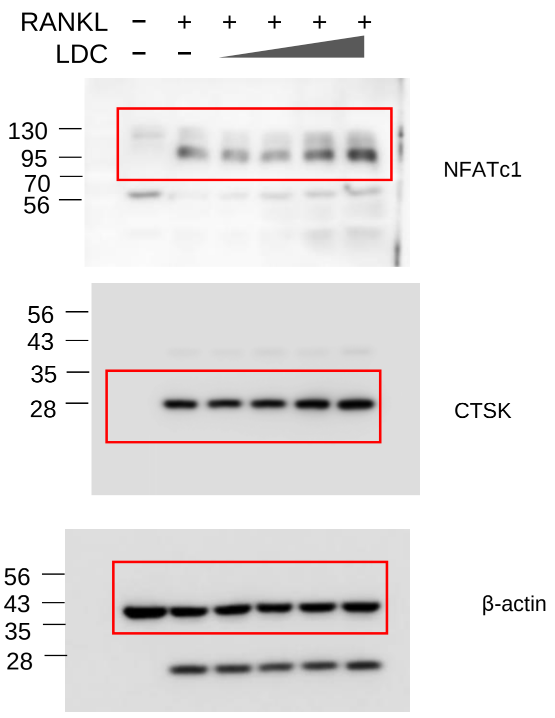

Fig. 3b

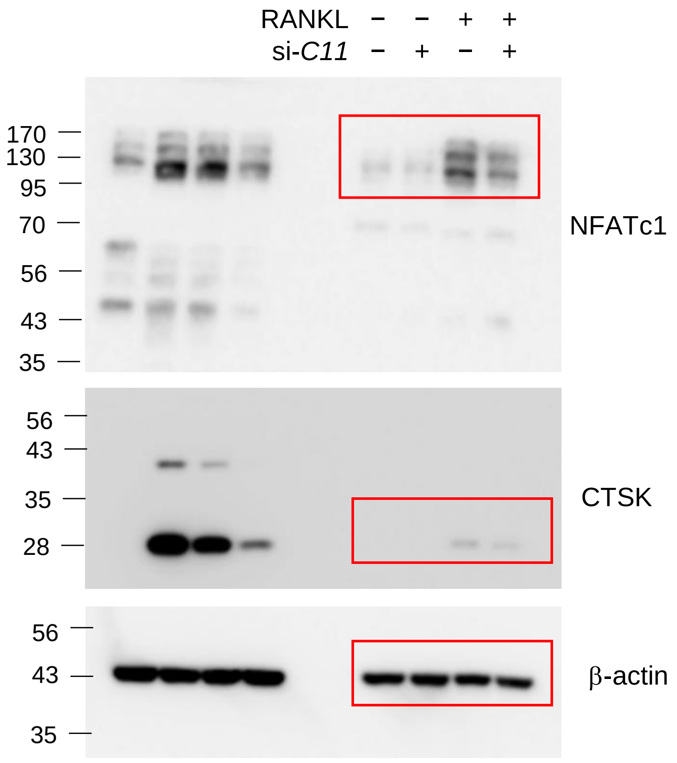

Fig. 3e

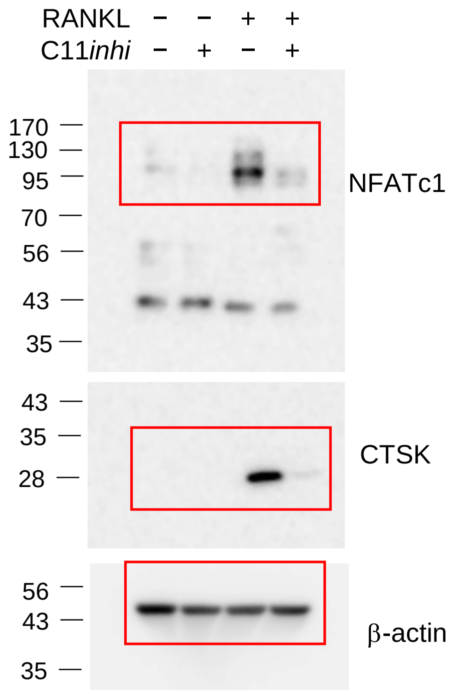

Fig. 3j

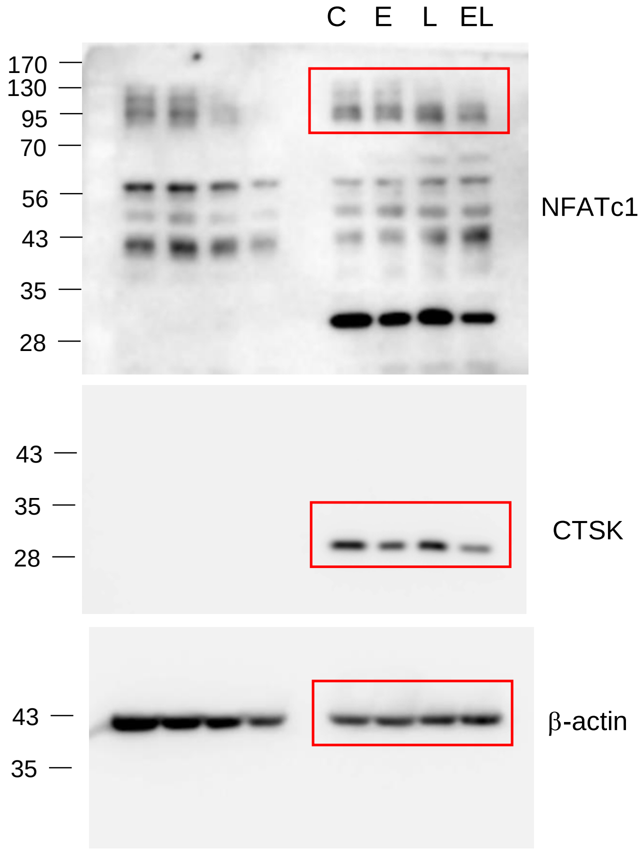

Fig. 5b

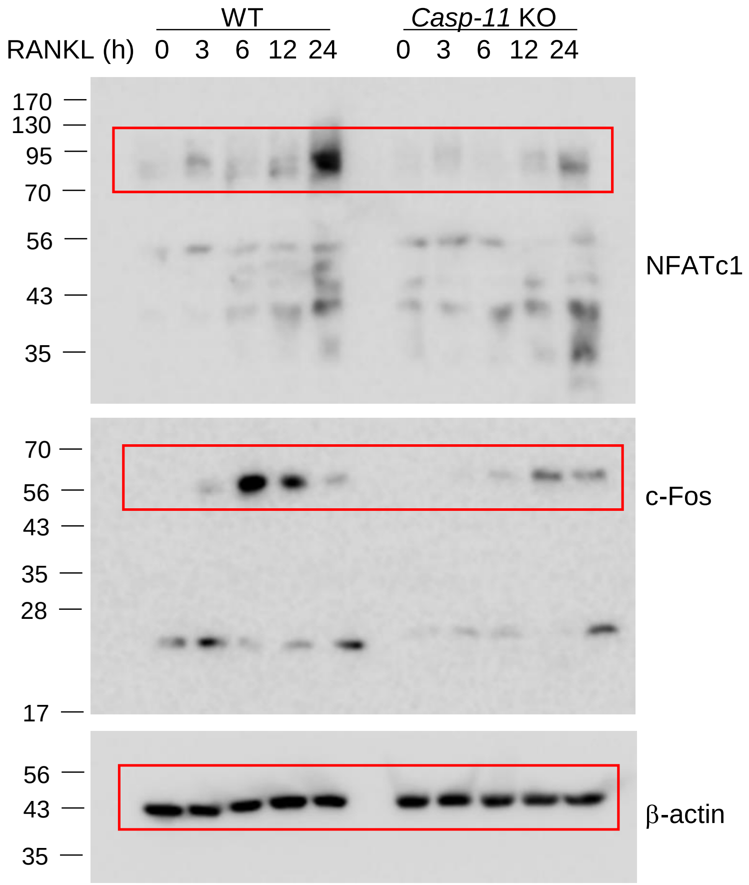
WT
Casp-11 KO
RANKL (h) 0 3 6 12 24 0 3 6 12 24
170
130
95
70
56
43
35
NFATc1
70
56
43
35
28
17
c-Fos
56
43
35
β-actin

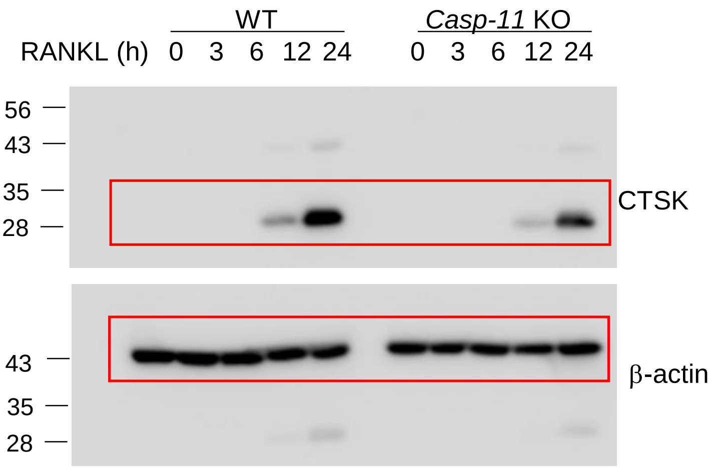
WT
Casp-11 KO
RANKL (h) 0 3 6 12 24 0 3 6 12 24
56
43
35
28
CTSK
43
35
28
β-actin

Fig. 5g

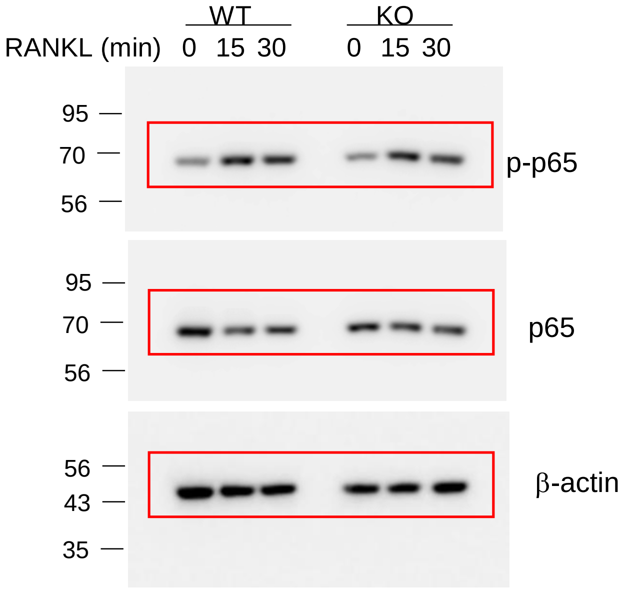
WT
KO
RANKL (min) 0 15 30 0 15 30
95
70
56
p-p65
95
70
56
p65
56
43
35
β-actin

Fig. 5h

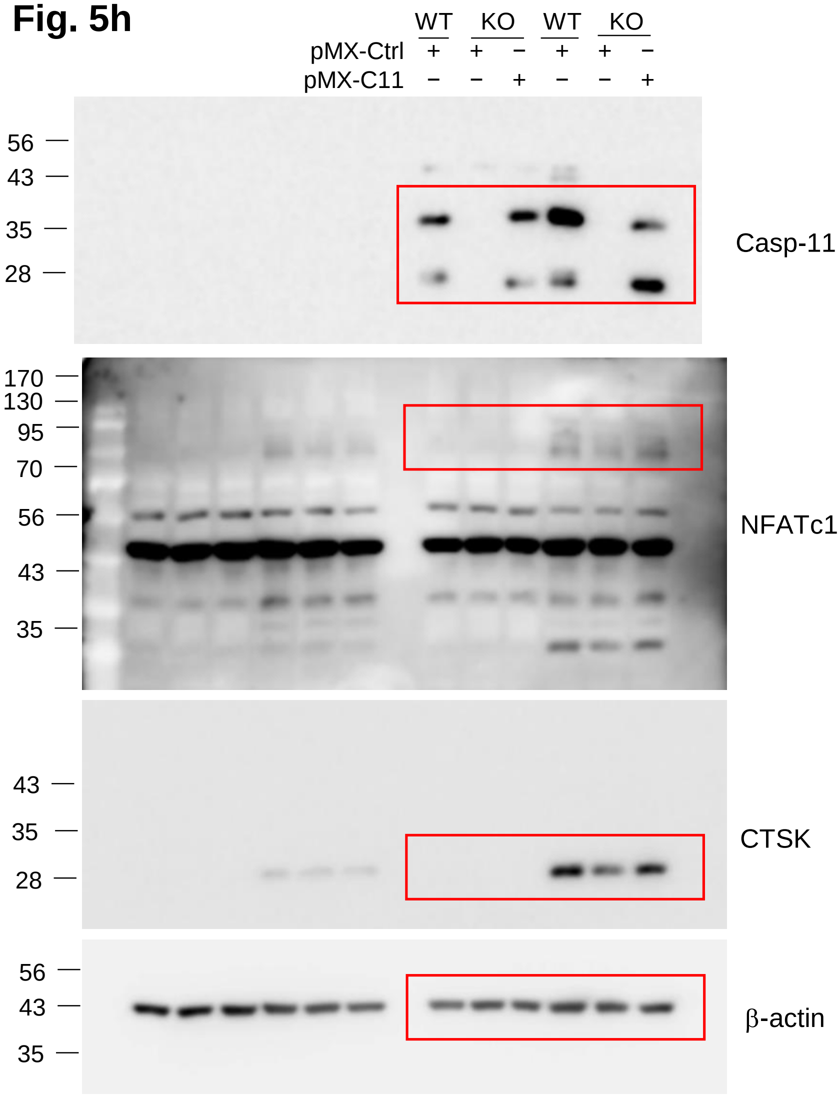
WT KO WT KO
pMX-Ctrl + + − + + −
pMX-C11 − − + − − +
56
43
35
28
Casp-11
170
130
95
70
56
43
35
NFATc1
43
35
28
CTSK
56
43
35
β-actin

Fig. 6a

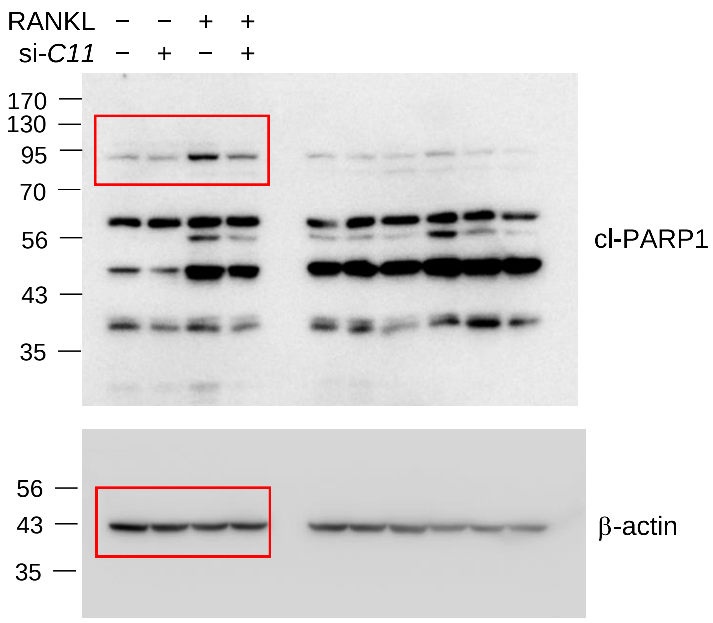

Fig. 6b

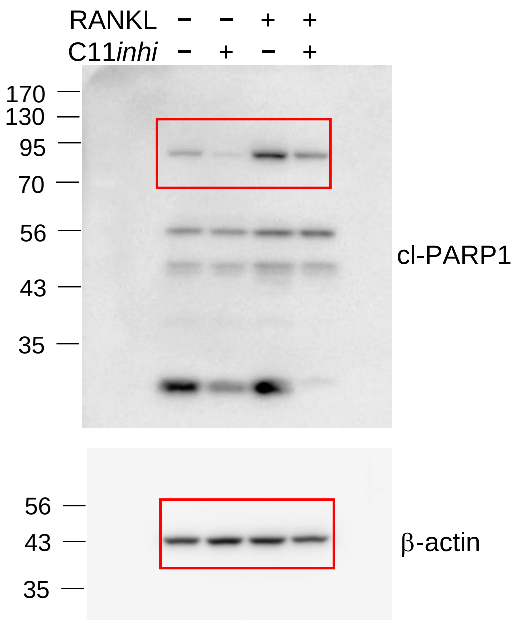

Fig. 6c

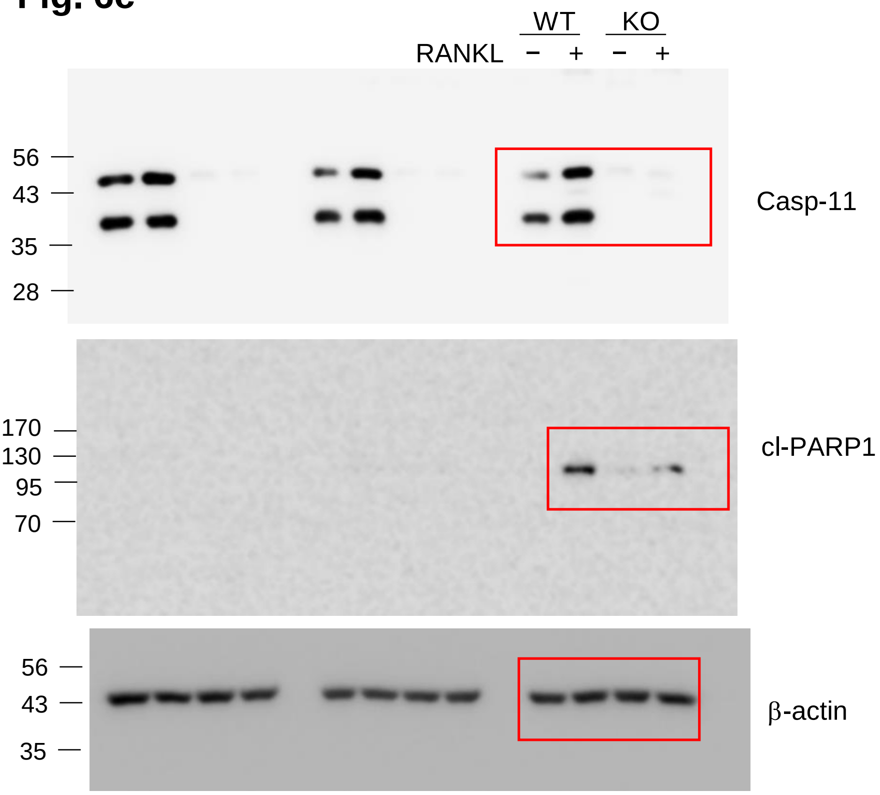

Fig. 6d

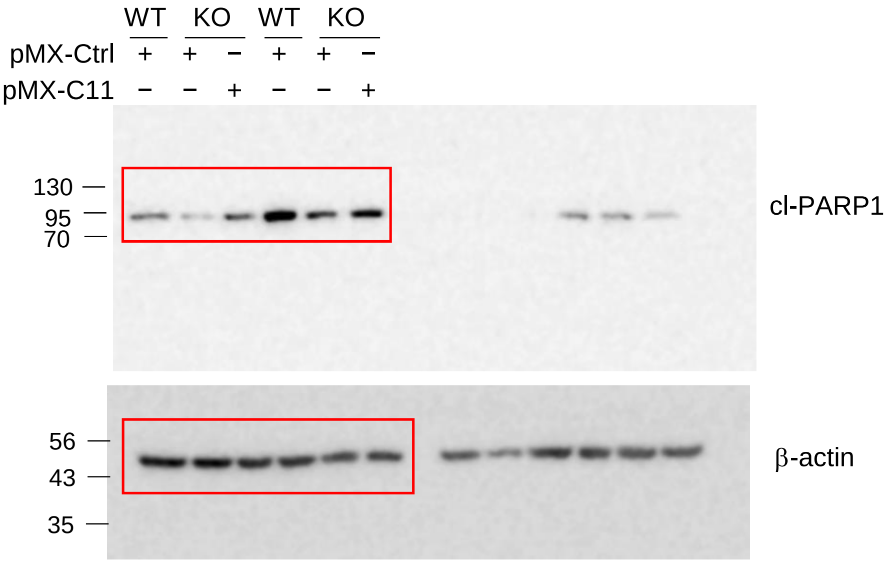

WT KO WT KO
pMX-Ctrl + + − + + −
pMX-C11 − − + − − +
130
95
70
cl-PARP1
56
43
35
β-actin

Fig. 6e

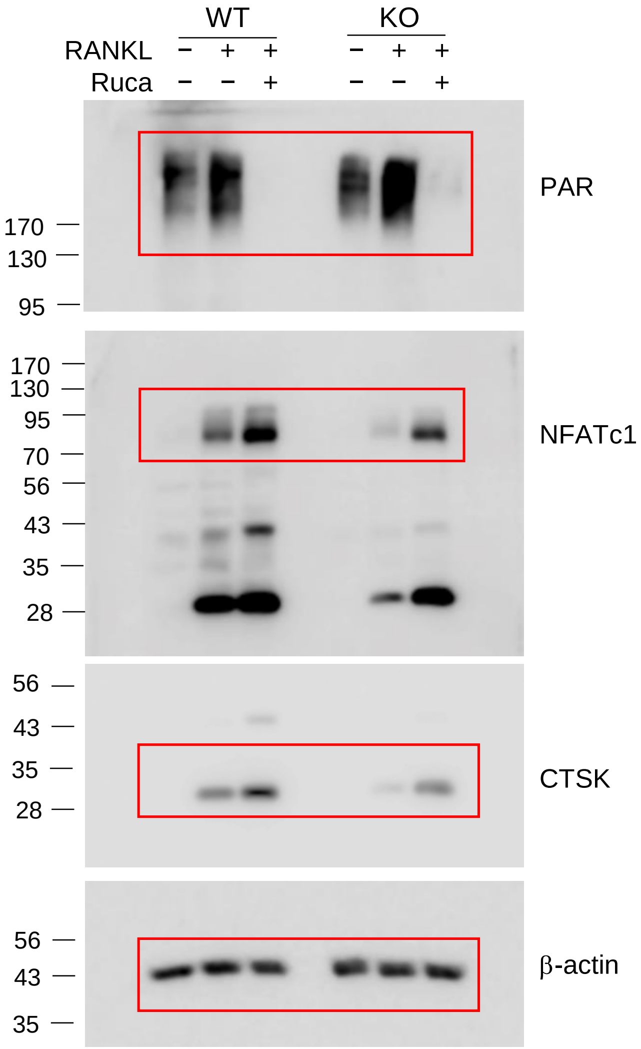

WT KO
RANKL − + + − + +
Ruca − − + − − +
170
130
95
PAR
170
130
95
70
56
43
35
28
NFATc1
56
43
35
28
CTSK
56
43
35
β-actin

Fig. 6h

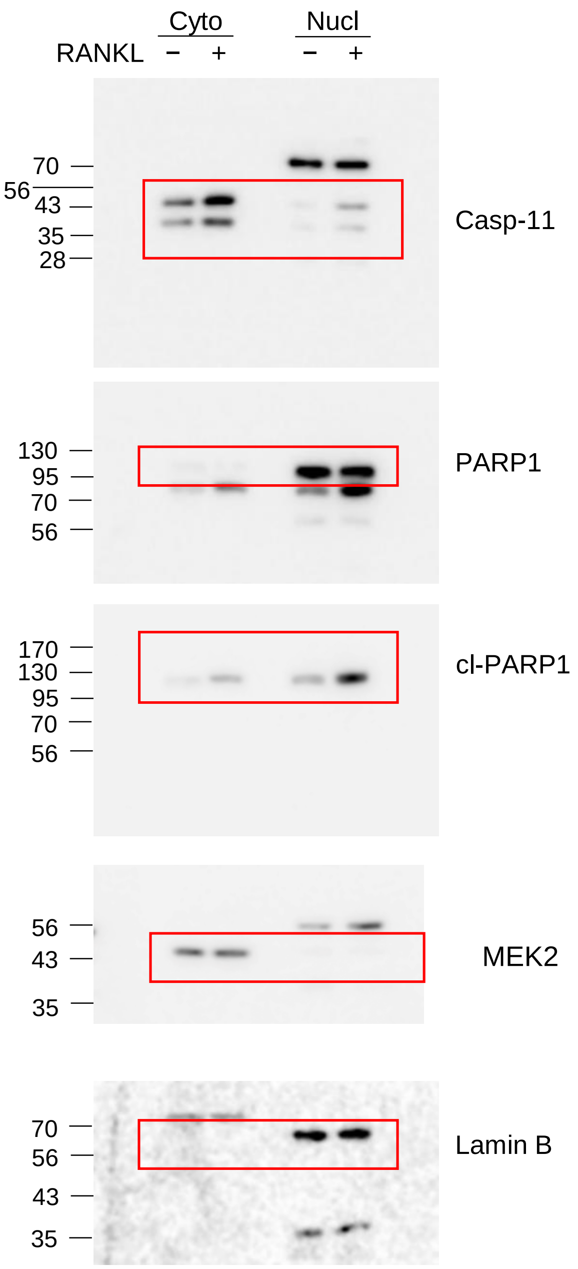

Cyto
Nucl
RANKL
−
+
−
+
70
56
43
35
28
Casp-11
130
95
70
56
PARP1
170
130
95
70
56
cl-PARP1
56
43
35
MEK2
70
56
43
35
Lamin B

Fig. 6k

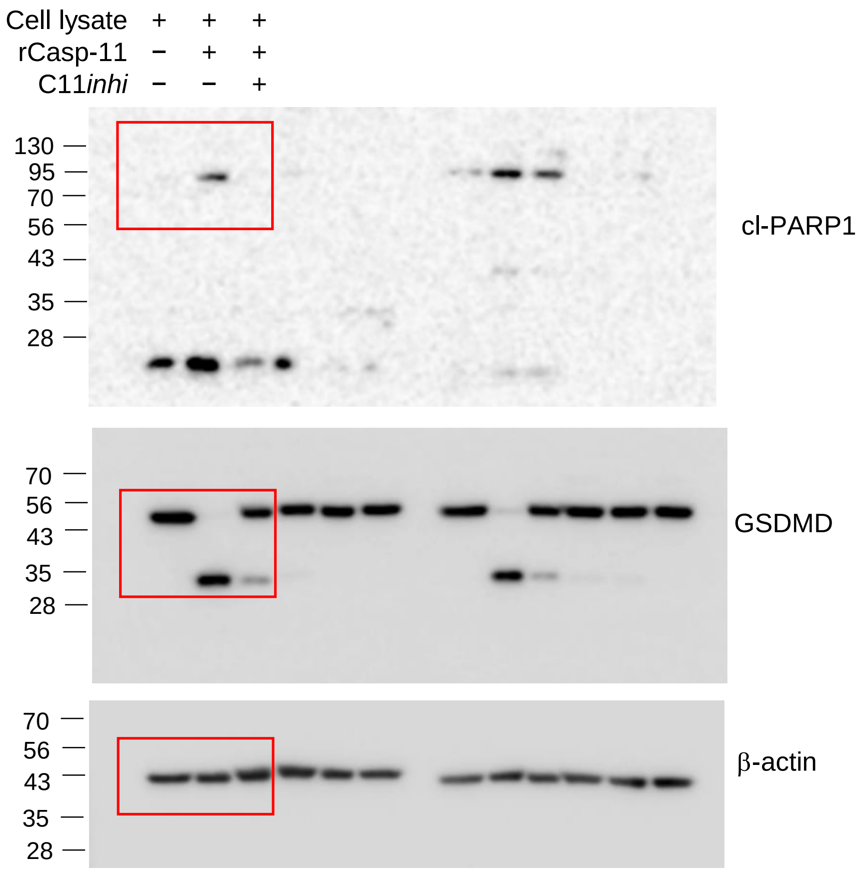

Cell lysate + + +
rCasp-11 − + +
C11*inhi* − − +
130
95
70
56
43
35
28
cl-PARP1
70
56
43
35
28
GSDMD
70
56
43
35
28
β-actin

Fig. 6l

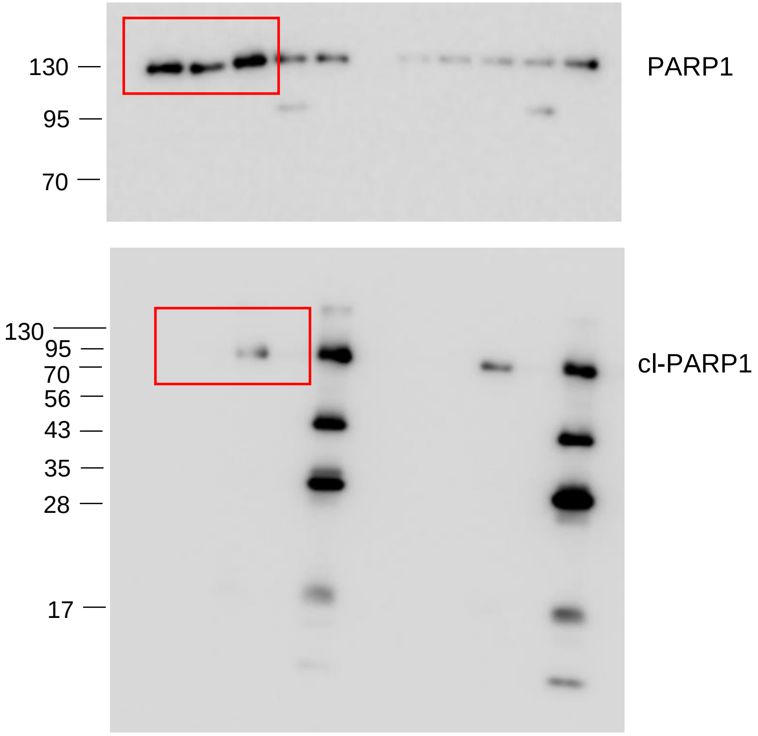

130
95
70
PARP1
130
95
70
56
43
35
28
17
cl-PARP1

Fig. 7a

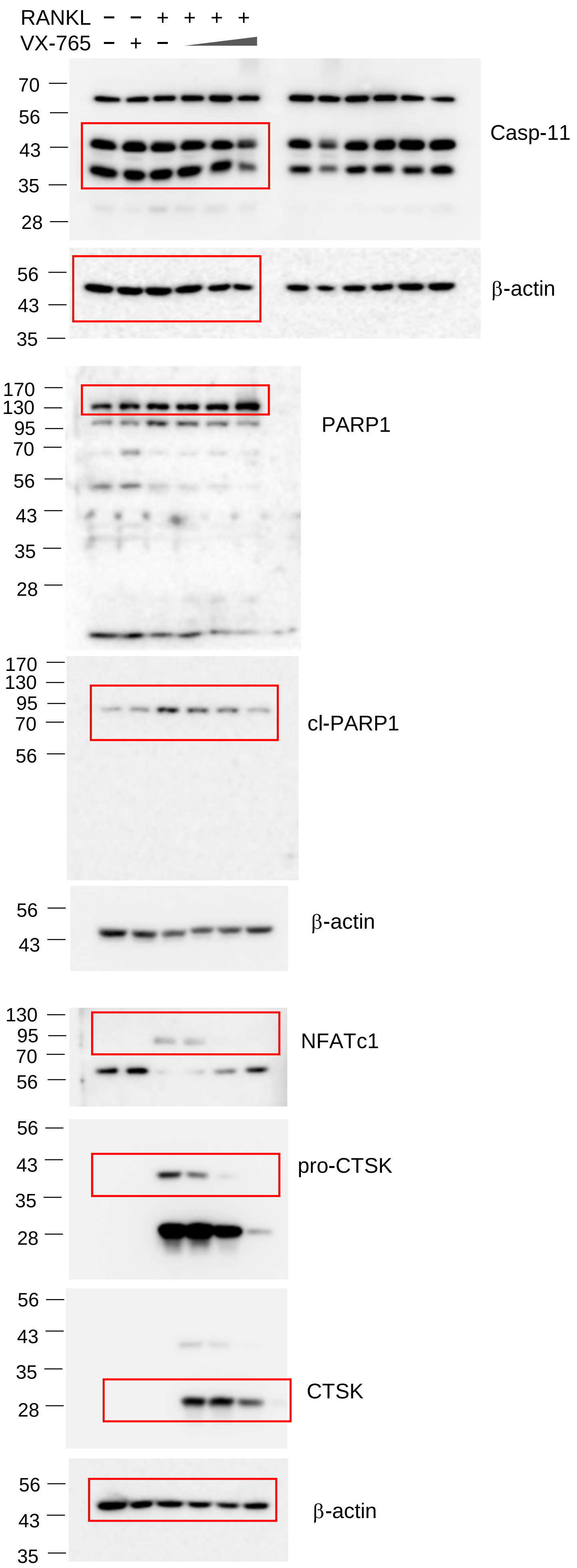
RANKL − − + + + +
VX-765 − + −
Casp-11
β-actin
PARP1
cl-PARP1
β-actin
NFATc1
pro-CTSK
CTSK
β-actin
70
56
43
35
28
170
130
95

S Fig. 1b

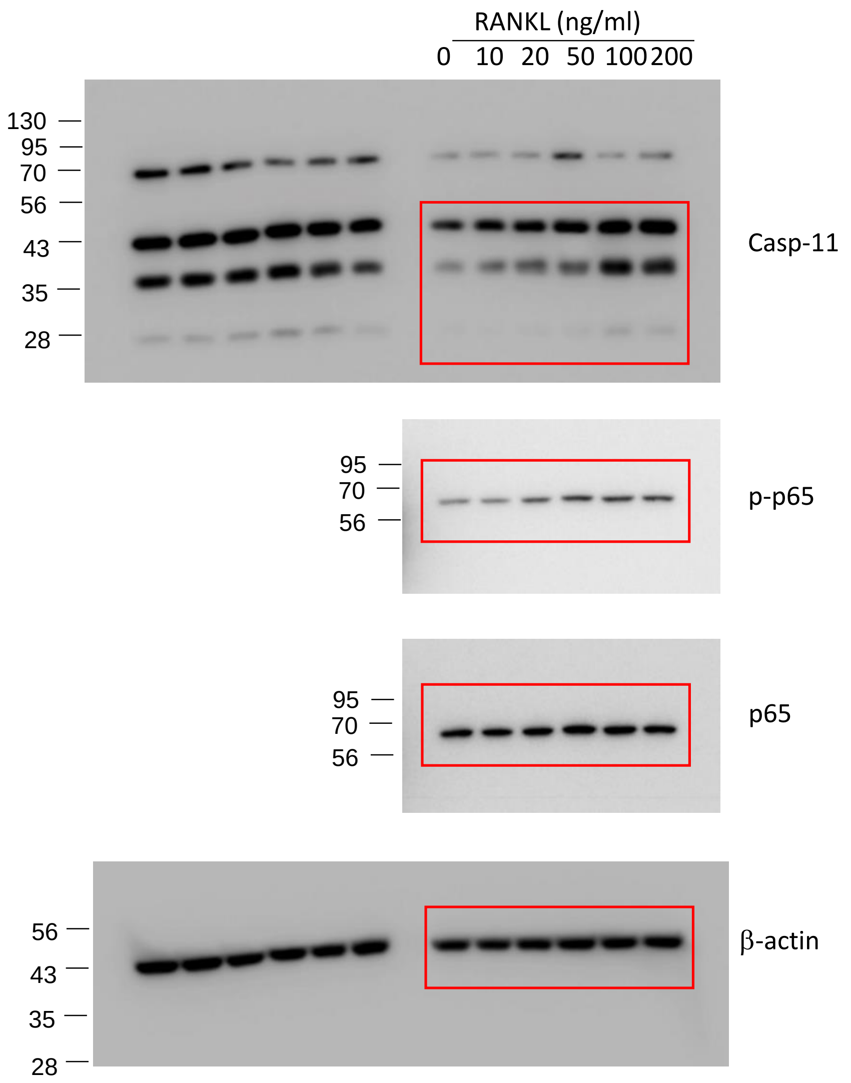

RANKL (ng/ml)
0 10 20 50 100 200
130
95
70
56
43
35
28
Casp-11
95
70
56
p-p65
95
70
56
p65
56
43
35
28
β-actin

S Fig. 2

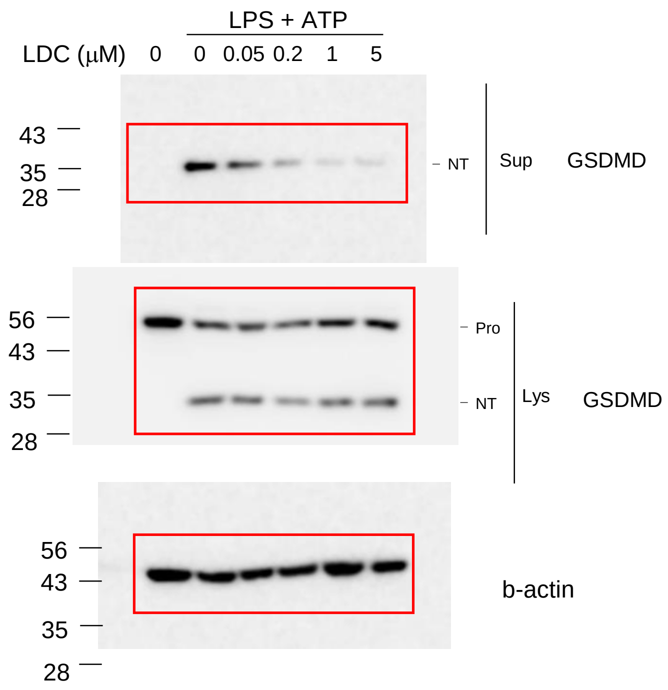
LPS + ATP
LDC (μM) 0 0 0.05 0.2 1 5
43
35
28
NT
Sup
GSDMD
56
43
35
28
Pro
NT
Lys
GSDMD
56
43
35
28
b-actin

S Fig. 3b

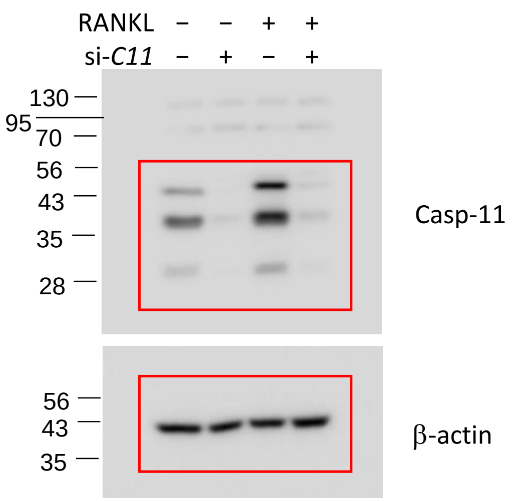

S Fig. 3c

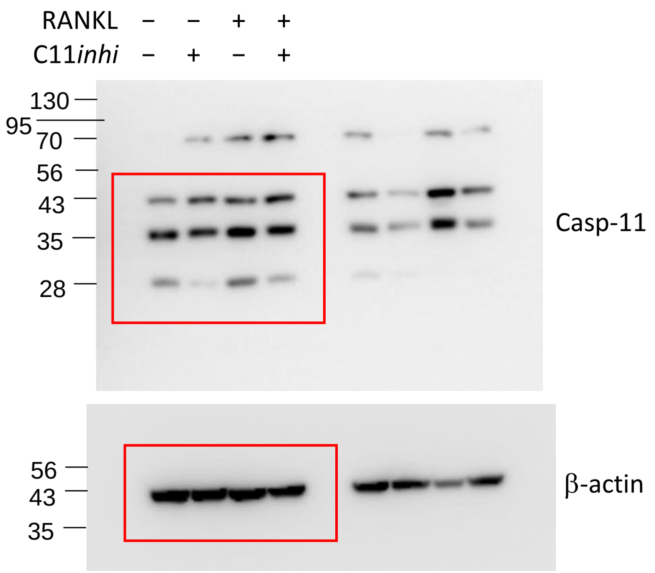

S Fig. 6b

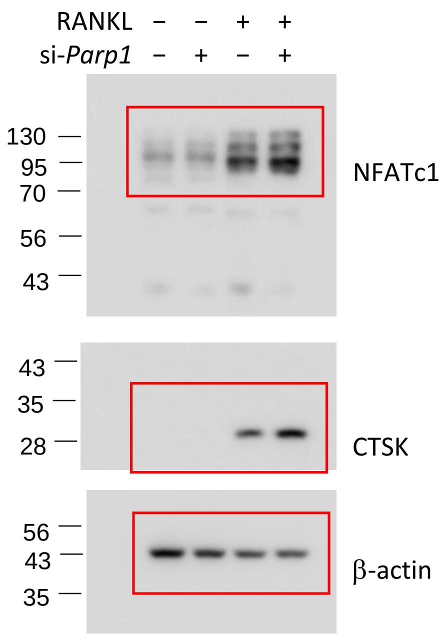

S Fig. 6e

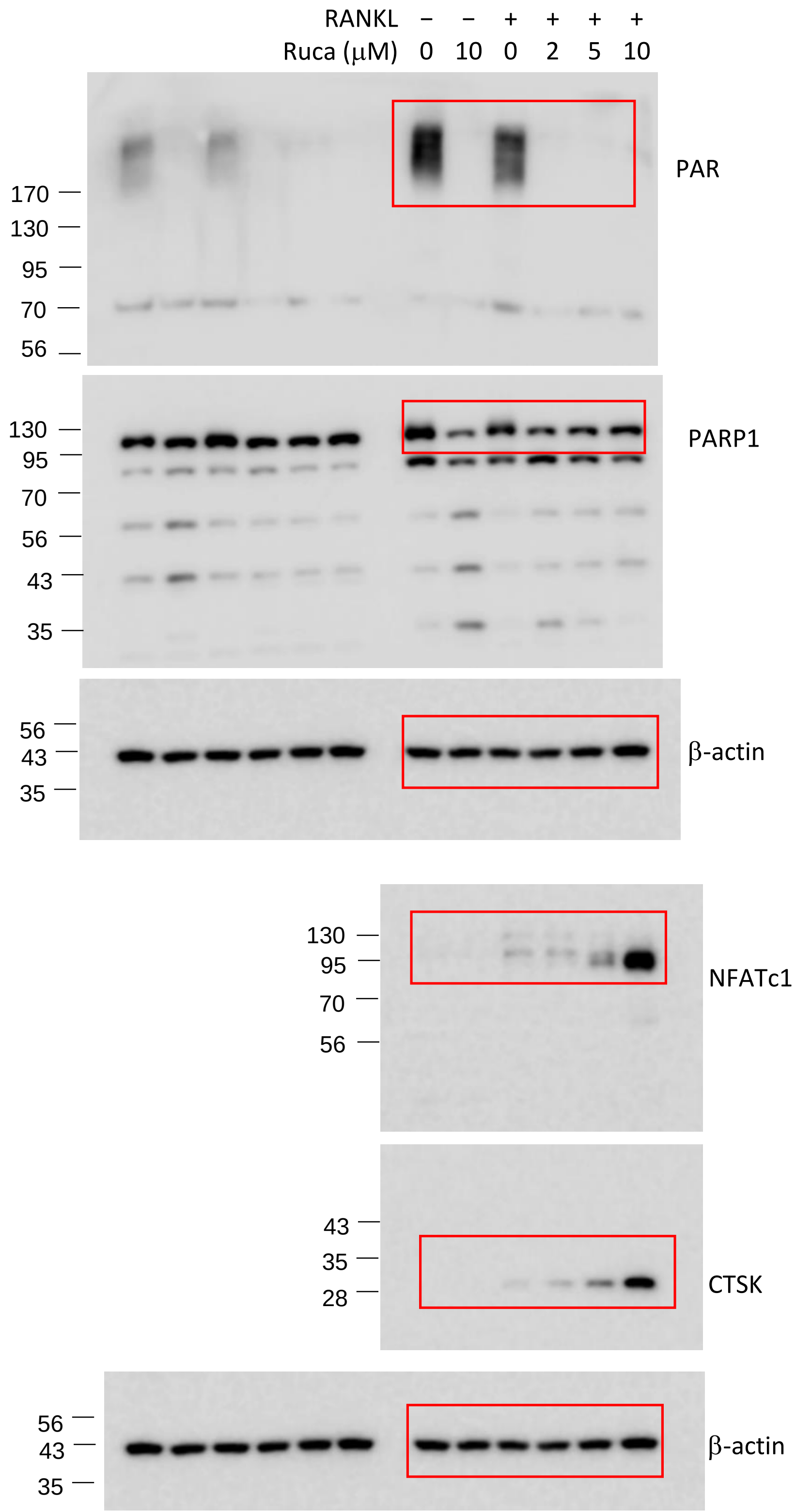

S Fig. 7b

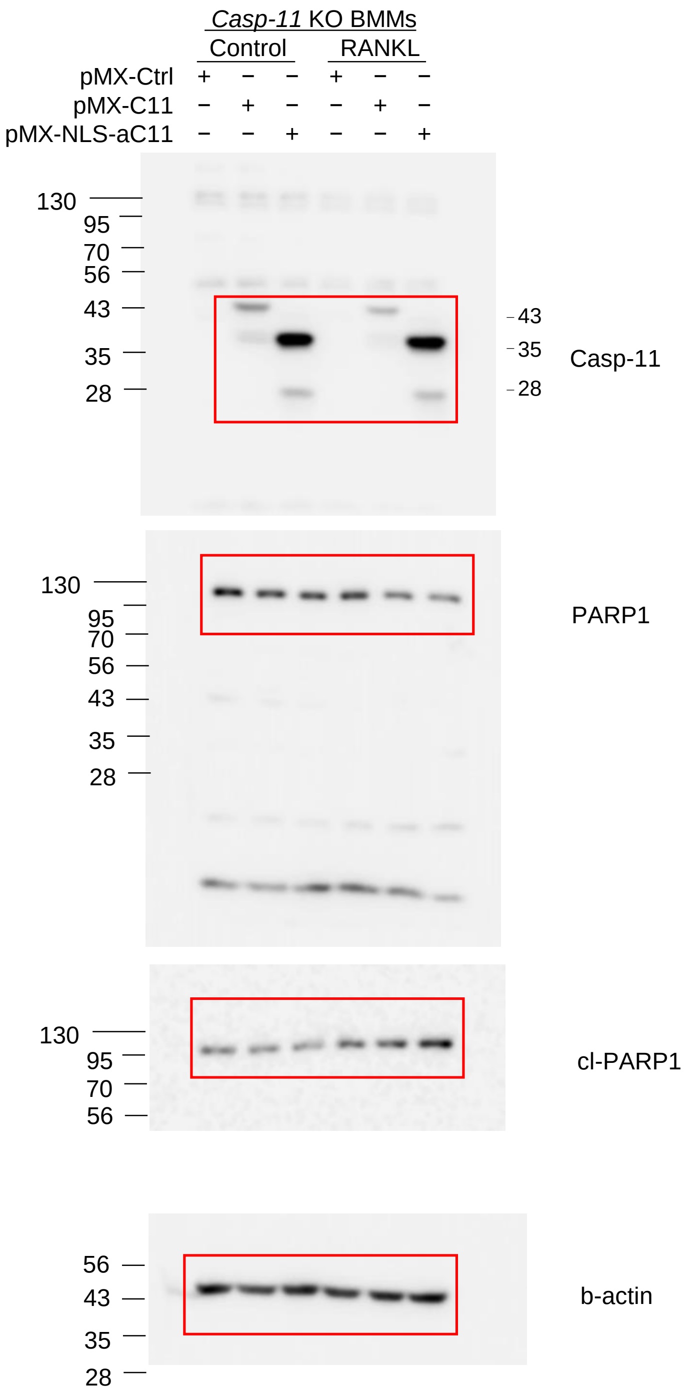

Casp-11 KO BMMs
Control
RANKL
pMX-Ctrl + − − + − −
pMX-C11 − + − − + −
pMX-NLS-aC11 − − + − − +
130
95
70
56
43
35
28
43
35
28
Casp-11
130
95
70
56
43
35
28
PARP1
130
95
70
56
cl-PARP1
56
43
35
28
b-actin

S Fig. 7c

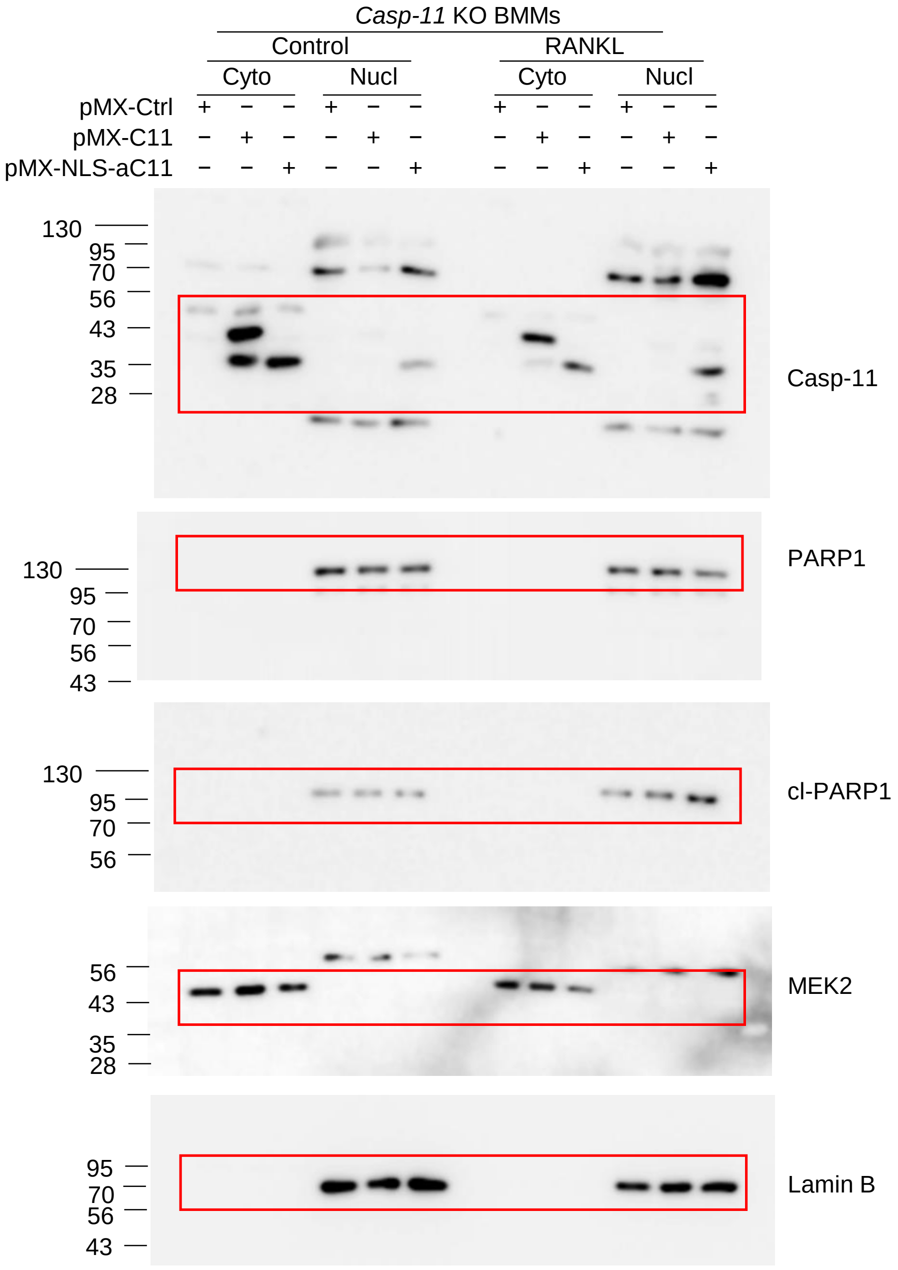
Casp-11 KO BMMs
Control
RANKL
Cyto
Nucl
Cyto
Nucl
pMX-Ctrl + − − + − − + − − + − −
pMX-C11 − + − − + − − + − − + −
pMX-NLS-aC11 − − + − − + − − + − − +
130
95
70
56
43
35
28
Casp-11
130
95
70
56
43
PARP1
130
95
70
56
cl-PARP1
56
43
35
28
MEK2
95
70
56
43
Lamin B

S Fig. 8a

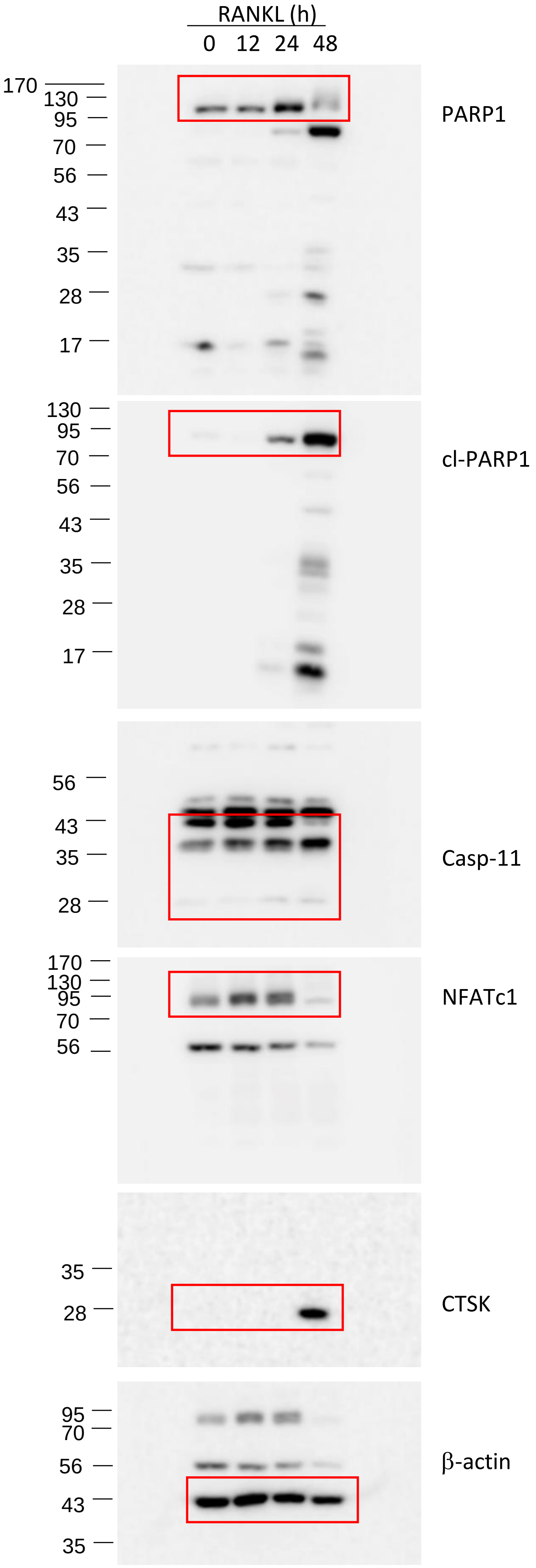

RANKL (h)
0 12 24 48
170
130
95
70
56
43
35
28
17
PARP1
130
95
70
56
43
35
28
17
cl-PARP1
56
43
35
28
Casp-11
170
130
95
70
56
NFATc1
35
28
CTSK
95
70
56
43
35
β-actin

S Fig. 8b

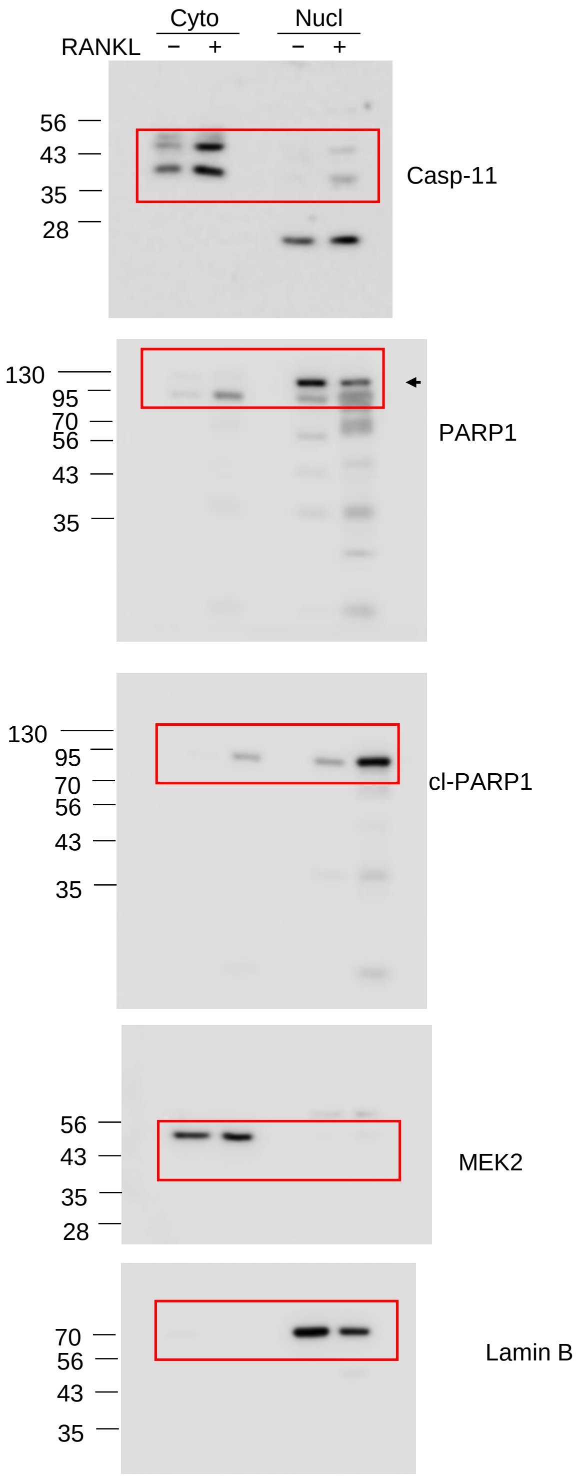
Cyto
Nucl
RANKL − + − +
56
43
35
28
Casp-11
130
95
70
56
43
35
PARP1
130
95
70
56
43
35
cl-PARP1
56
43
35
28
MEK2
70
56
43
35
Lamin B

S Fig. 9b

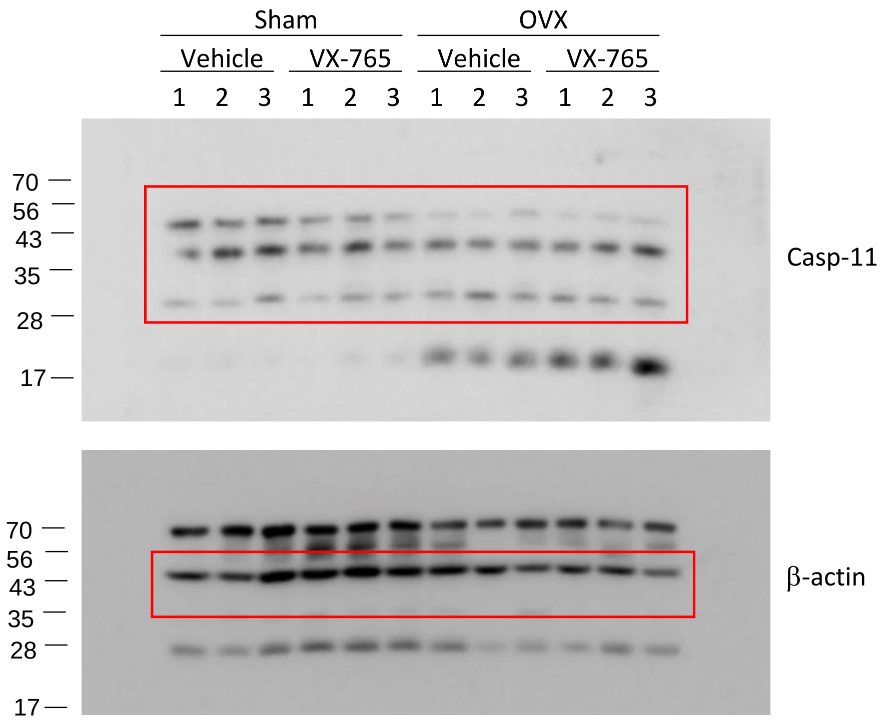
Sham
OVX
Vehicle
VX-765
Vehicle
VX-765
1 2 3 1 2 3 1 2 3 1 2 3
70
56
43
35
28
17
Casp-11
70
56
43
35
28
17
β-actin
